# Cancer Nanobombs Delivering Artoxplatin with a Polyigniter Bearing Hydrophobic Ferrocene Units Upregulate PD‐L1 Expression and Stimulate Stronger Anticancer Immunity

**DOI:** 10.1002/advs.202300806

**Published:** 2023-05-11

**Authors:** Yongchao Gao, Hanchen Zhang, Lin Tang, Feifei Li, Li Yang, Haihua Xiao, Johannes Karges, Weihua Huang, Wei Zhang, Chaoyong Liu

**Affiliations:** ^1^ Department of Clinical Pharmacology Xiangya Hospital Central South University 87 Xiangya Road Changsha 410008 P. R. China; ^2^ Institute of Clinical Pharmacology Central South University Hunan Key Laboratory of Pharmacogenetics 110 Xiangya Road Changsha 410078 P. R. China; ^3^ Engineering Research Center of Applied Technology of Pharmacogenomics Ministry of Education 110 Xiangya Road Changsha 410078 P. R. China; ^4^ National Clinical Research Center for Geriatric Disorders 87 Xiangya Road Changsha Hunan 410008 P. R. China; ^5^ Beijing National Laboratory for Molecular Sciences Key Laboratory of Polymer Physics and Chemistry and CAS Key Laboratories of Organic Solids Institute of Chemistry Chinese Academy of Sciences Beijing 100190 P. R. China; ^6^ University of Chinese Academy of Sciences Beijing 100049 P. R. China; ^7^ Beijing Advanced Innovation Center for Soft Matter Science and Engineering College of Life Science and Technology Beijing University of Chemical Technology Beijing 100029 P. R. China; ^8^ Institute of Chinese Medical Sciences State Key Laboratory of Quality Research in Chinese Medicine University of Macau Macao 999078 P. R. China; ^9^ Faculty of Chemistry and Biochemistry Ruhr‐University Bochum Universitätsstrasse 150 44780 Bochum Germany; ^10^ Hunan Provincial Tumor Hospital and the Affiliated Tumor Hospital of Xiangya Medical School Central South University Changsha 410006 P. R. China; ^11^ Key Specialty of Clinical Pharmacy The First Affiliated Hospital of Guangdong Pharmaceutical University Guangzhou 510080 P. R. China

**Keywords:** artoxplatin, chemo‐immunotherapy, immunogenic cell death, oxaliplatin, reactive oxygen species

## Abstract

Poor immunogenicity seriously hampers the broader implementation of antitumor immunotherapy. Enhanced immunogenicity capable of achieving greater antitumor immunity is urgently required. Here, a novel polymer that contains hydrophobic ferrocene (Fc) units and thioketal bonds in the main chain, which further delivered a prodrug of oxaliplatin and artesunate, i.e., Artoxplatin, to cancer cells is described. This polymer with Fc units in the nanoparticle can work as a polyigniter to spark the peroxide bonds in Artoxplatin and generate abundant reactive oxygen species (ROS) to kill cancers as nanobomb^ig^ for cancer therapy. Moreover, ROS can trigger the breakdown of thioketal bonds in the polymer, resulting in the biodegradation of the polymer. Importantly, nanobomb^ig^ can facilitate the maturation of dendritic cells and promote the activation of antitumor immunity, through the enhanced immunogenic cell death effect by ROS generated in situ. Furthermore, metabolomics analysis reveals a decrease in glutamine in nanobomb^ig^‐treated cancer cells, resulting in the upregulation of programmed death ligand 1 (PD‐L1). Consequently, it is further demonstrated enhanced tumor inhibitory effects when using nanobomb^ig^ combined with anti‐PD‐L1 therapy. Overall, the nanosystem offers a rational design of an efficient chemo‐immunotherapy regimen to promote antitumor immunity by improving tumor immunogenicity, addressing the key challenges cancer immunotherapy faced.

## Graphical abstract

A strategy for promoting chemo‐immunotherapy by simultaneously delivering oxaliplatin and artesunate into the tumor using a nanoparticle containing ferrocene units, named nanobomb^ig^, which facilitates the maturation of dendritic cells and promotes the activation of antitumor immune response, through the enhanced immunogenic cell death effect by reactive oxygen species generated in situ.

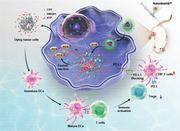

## 1 Introduction

Cancer immunotherapy has attracted increasing attention in cancer therapy.^[^ ^1^ ^]^ Despite the great advances in immunotherapy, insufficient response rates of patients still hinder its clinical application.^[^ ^2^ ^]^ To improve response rates, traditional radiotherapy and chemotherapy are typically combined with immunotherapy in the clinic.^[^ ^3^ ^]^ However, these traditional treatments may result in immunosuppression, making things even worse with lower response rates to immunotherapy.^[^ ^4^ ^]^ Recent studies have indicated that poor immunogenicity^[^ ^5^ ^]^ and inefficient maturation of dendritic cells (DCs)^[^ ^6^ ^]^ play important roles in low response to cancer therapy. Therefore, enhancing immunogenicity at the tumor site and promoting the maturation of antigen‐presenting cells (APCs) are essential for activating tumor immunity.^[^ ^7^ ^]^

Recently, numerous studies have shown that the immunogenicity of tumor cells could be induced by the death of tumor cells, which is called immunogenic cell death (ICD).^[^ ^8^ ^]^ During this process, a series of signaling molecules known as damage‐associated molecular patterns (DAMPs) are released, including calreticulin (CRT), adenosine triphosphate (ATP), and high mobility group protein B1 (HMGB1).^[^ ^9^ ^]^ Furthermore, the released DAMPs work as endogenous “dangerous” signals^[^ ^10^ ^]^ to increase the phagocytosis of tumor antigens by DCs, thus facilitating DCs maturation, antigen representation, and robust antitumor immune response.^[^ ^8^ ^]^

Oxaliplatin (Oxa) is the third‐generation platinum cytotoxic drug,^[^ ^11^ ^]^ which induces not only apoptosis of cancer cells, but also the ICD effect to stimulate antitumor immune responses.^[^ ^12^ ^]^ However, the key bottleneck of Oxa is that it cannot trigger enough ICD effects to eliminate tumors. With the in‐depth study, researchers have found that cancer therapy strategies such as photodynamic therapy (PDT) can induce a large number of reactive oxygen species (ROS) in tumor cells, increasing oxidative stress to induce the ICD effect.^[^ ^8^, ^13^ ^]^ However, the generation of ROS induced by PDT relies on light excitation, but the penetration depth and transmission efficiency are limited.^[^ ^14^ ^]^ Artesunate (ART) is commonly used in antimalarial treatment, which could generate highly toxic ROS via a Fenton‐like reaction with intracellular iron, independent of exogenous stimuli.^[^ ^15^ ^]^ The above findings remind us of the possibility of developing effective therapeutic strategies that can efficiently generate ROS independent of external stimuli to enhance the ICD effect and subsequent antitumor immunity.

**Scheme 1:**
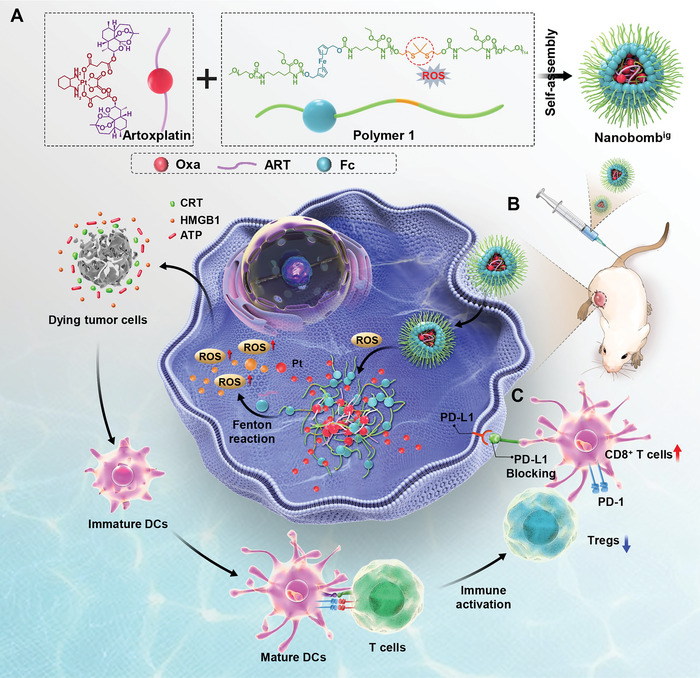
Schematic illustration of the cancer nanobomb^ig^ for upregulating PD‐L1 expression and stimulating stronger immune response. A) Artoxplatin was encapsulated by the ROS responsive polyigniters (Polymer1) with Fc units and thioketal bonds in the main chain to form nanobomb^ig^. B) After *i.v*. injection, nanobomb^ig^ was efficiently accumulated at the tumor site, and was internalized by the cancer cells. On one hand, Artoxplatin can be reduced in cancer cells to release toxic Oxa and ART. On the other hand, the released ART was catalyzed to generate ROS, which then increased the release of DAMPs by dying tumor cells, and amplified the ICD effect of Oxa and facilitated immune activation. C) Nanobomb^ig^ up‐regulated the expression of PD‐L1, and exerted a synergistic antitumor effect by combined with PD‐L1 mAb.

Herein, a platinum (IV) (Pt(IV)) prodrug of Oxa with two ART molecules in the axial positions was designed (designated as Artoxplatin, **Scheme** **1**A). Artoxplatin can be reduced in cancer cells to release Oxa and ART. Notably, on the one hand, the released Oxa can result in apoptosis and cell death as well as the ICD effect. On the other hand, ART can be catalyzed by intracellular Fe^2+^/Fe^3+^ via Fenton reaction, to generate a greater number of oxygen radicals. Subsequently, these free radicals damage DNA, proteins, lipids, etc., within cancer cells, thereby killing cancer cells. Therefore, artoxplatin can actually be further considered as a cancer bomb, and iron ions in cells can be regarded as an igniter.

To further enhance the efficacy of Artoxplatin, an amphiphilic triblock polymer containing thioketal bonds and hydrophobic ferrocene (Fc) units in the polymer backbone was designed. This polymer was sensitive to ROS, which can be broken down to release Fc at the +2 valence oxidation state. Further, Fc can be oxidized into hydrophilic Fc at the +3 valence oxidation state, which could catalyze ART to generate more radicals by the Fenton reaction. Therefore, this polymer can be deemed as an exogenous polyigniter. Based on this, the encapsulation of Artoxplatin with the polyigniter results in the nanobomb^ig^ capable of delivering both the cancer bomb of Artoxplatin and polyigniter simultaneously into tumor cells, ensuring delivery efficiency. ^[^ ^16^ ^]^ A polymer containing thioketal bonds without Fc was also designed to deliver Artoxplatin as a nanobomb. The nanobomb^ig^ was evaluated both in vitro and in vivo in different cancer cell lines and the CT26 colorectal cancer animal model to demonstrate the effectiveness of delivering both the tumor bombs and polyigniters into cancer cells to activate tumor immunity (Scheme 1B). Moreover, we found for the first time that the nanobomb^ig^ had no immunosuppressive effect, and could even increase the expression of programmed death ligand 1 (PD‐L1) by inhibiting glutamine utilization through a metabolomics study. The new findings paved the way for its combination with immune checkpoint blockade therapy of PD‐L1 mono antibodies (mAb) in vivo (Scheme 1C). Taken together, the results showed that the self‐assembled nanobomb^ig^ could amplify the ICD effect, facilitate DCs maturation, and finally elicit robust antitumor immunity. Furthermore, nanobomb^ig^ combined with PD‐L1 mAb significantly inhibited the growth of CT26 subcutaneous tumors and exhibited a powerful antitumor immune effect.

## 2 Results and Discussion

### 2.1 ART Increases ROS Generation and Enhances the ICD Effect of Oxa

**Figure 1:**
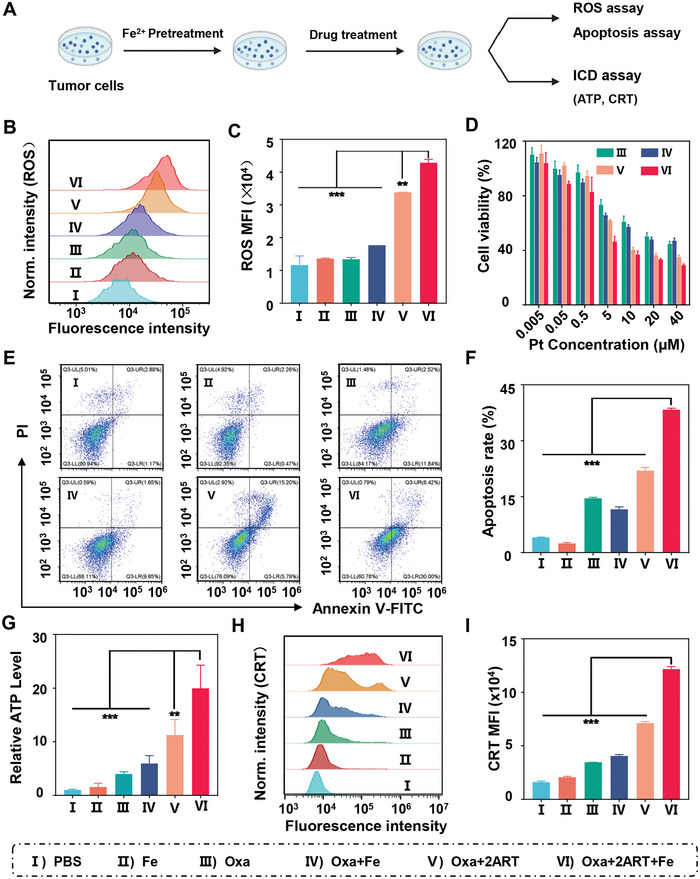
ART enhances the anticancer efficacy and ICD effect of Oxa, which can be further promoted by Fe^2+^ pretreatment. A) Schematic illustration of the pretreatment of CT26 cells with Fe^2+^ and combination of ART with Oxa for enhancing ICD. B,C) ROS generation and its semiquantification by flow cytometry (FCM) in CT26 cells treated with various compounds at 7 h (25 µm Pt, 50 µm ART). D) Representative cell viability of CT26 cells treated with various compounds at 48 h. E) FCM images and F) Apoptosis rates of CT26 cells treated with various compounds at 48 h (5 µm Pt, 10 µm ART). G) Relative ATP levels in CT26 cells treated with various compounds (5 µm Pt, 10 µm ART). H,I) Flow cytometric analysis of CRT and its corresponding semiquantification (25 µm Pt, 50 µm ART). CT26 cells were pretreated with or without Fe^2+^ at 10 µm for 10 h. Data are presented as mean ± SD. Statistical significances between every two groups were calculated via one‐way ANOVA. **p < 0.01, ***p < 0.001.

To test the hypothesis that ART could increase intracellular ROS and enhance Oxa induced ICD effect, we conducted a series of experiments in vitro (**Figure** **1**A). Since ART could enhance the generation of ROS via the opening of endoperoxide bridges,^[^ ^15a^ ^]^ a fluorescent probe of 2', 7'‐dichlorodihydrofluorescein diacetate (DCHF‐DA) was adopted to probe ROS in CT26 cells treated with ART. The results demonstrated that Oxa+2ART resulted in nearly 2.5‐fold higher fluorescence intensity than Oxa in CT26 cells (Figure 1B,C). It is reported that Fe^2+^ could open the peroxide bridge of ART to generate oxygen radicals via Fenton chemistry.^[^ ^17^ ^]^ Subsequently, we demonstrated that the pretreatment of CT26 cells with Fe^2+^ enhanced the ROS generation by 1.3‐fold in cells treated with Oxa+2ART compared to those without Fe^2+^ pretreatment (Figure 1B,C). The cytotoxicity of various compounds was studied by the MTT assay in CT26, MC38, and HCT116 cell lines. The results demonstrated that Oxa+2ART showed a significantly better cell‐killing effect than Oxa in CT26 cells (IC_50_ = 3.06 µm vs IC_50_ = 5.65 µm in CT26, IC_50_: half‐inhibitory concentration) (Figure 1D). Moreover, if CT26 cells were pretreated with Fe^2+^, Oxa+2ART was much more potent with an IC_50_ value only at 1.82 µm (Figure 1D). Similar results were observed in MC38 and HCT116 cell lines (Figure S1, Supporting Information). The subsequent apoptosis assay indicated that Oxa+2ART induced an apoptosis rate of 22.51% on CT26 cells, which was ≈1.5 times that of cells treated with Oxa (14.56%). Furthermore, the apoptosis rate induced by Oxa+2ART increased to 38.36% when the CT26 cells were pretreated with Fe^2+^ (Figure 1E,F).

Oxa was reported to induce ICD effect by increasing the release of DAMPs from dying cells.^[^ ^8^, ^18^ ^]^ Moreover, ART can generate ROS, which is possible to substantially enhance ICD. To verify this hypothesis, the released DAMPs were studied including the secreted ATP as a “find me” signal and the exposed CRT as “eat me” signal.^[^ ^19^ ^]^ The released ATP can be quantified by an ATP assay kit, whereas the CRT can be quantified by FCM. The results showed that the released ATP in the supernatant of CT26 cells treated with Oxa+2ART was almost 1.8 times higher than that of Oxa (Figure 1G). Moreover, the fluorescence intensity of CRT in CT26 cells treated with Oxa+2ART was about 2.1 times higher than that of Oxa (Figure 1H,I, Supporting Information). Furthermore, the released ATP and CRT expression triggered by Oxa+2ART were further increased in Fe^2+^‐saturated CT26 cells, reaching up to 5.0‐fold and 3.5‐fold times higher than those in Oxa‐treated cells, respectively (Figure 1G–I). Taken together, the above findings indicated that Oxa+2ART induced stronger cytotoxicity and ICD effects, and these effects could be further enhanced with Fe^2+^ pretreatment.

### 2.2 Design and Anticancer Activity of Artoxplatin

Artoxplatin was prepared by oxidation of Oxa, and subsequent esterification of ART (Figure S2A, Supporting Information). The successful synthesis of Artoxplatin was confirmed by ^1^H NMR, ^13^C NMR, ^195^Pt‐NMR, and HR‐ESI‐MS, respectively (Figures S2–S4, Supporting Information). The existence of Pt^IV^ was confirmed by the ^195^Pt‐NMR signal at ≈1617.02 ppm (Figure S3, Supporting Information). The HR‐ESI‐MS spectrum gave a major peak at *m/z* 1186.39 081 assignable to [*M*+Na]^+^ (Figure S4, Supporting Information). A purity at 97.84% was found for Artoxplatin by high performance liquid chromatography (HPLC) (Figure S5, Supporting Information). The ROS generation capacity of Artoxplatin was examined in CT26 cells. The FCM results indicated that Artoxplatin generated comparable ROS to that of Oxa+2ART. Moreover, the ROS generation was further enhanced in Fe^2+^ pretreated cells for the treatment groups with Artoxplatin (Figure S6, Supporting Information). Finally, Artoxplatin had a cytotoxicity comparable to Oxa+2ART by MTT assay and an apoptosis assay (Figures S7 and S8, Supporting Information). More importantly, the released ATP and CRT expression in CT26 cells treated with Artoxplatin were similar to those treated with Oxa+2ART (Figure S9, Supporting Information).

### 2.3 Design of Polyigniter, Preparation, and Characterization of Nanobomb^ig^

**Figure 2:**
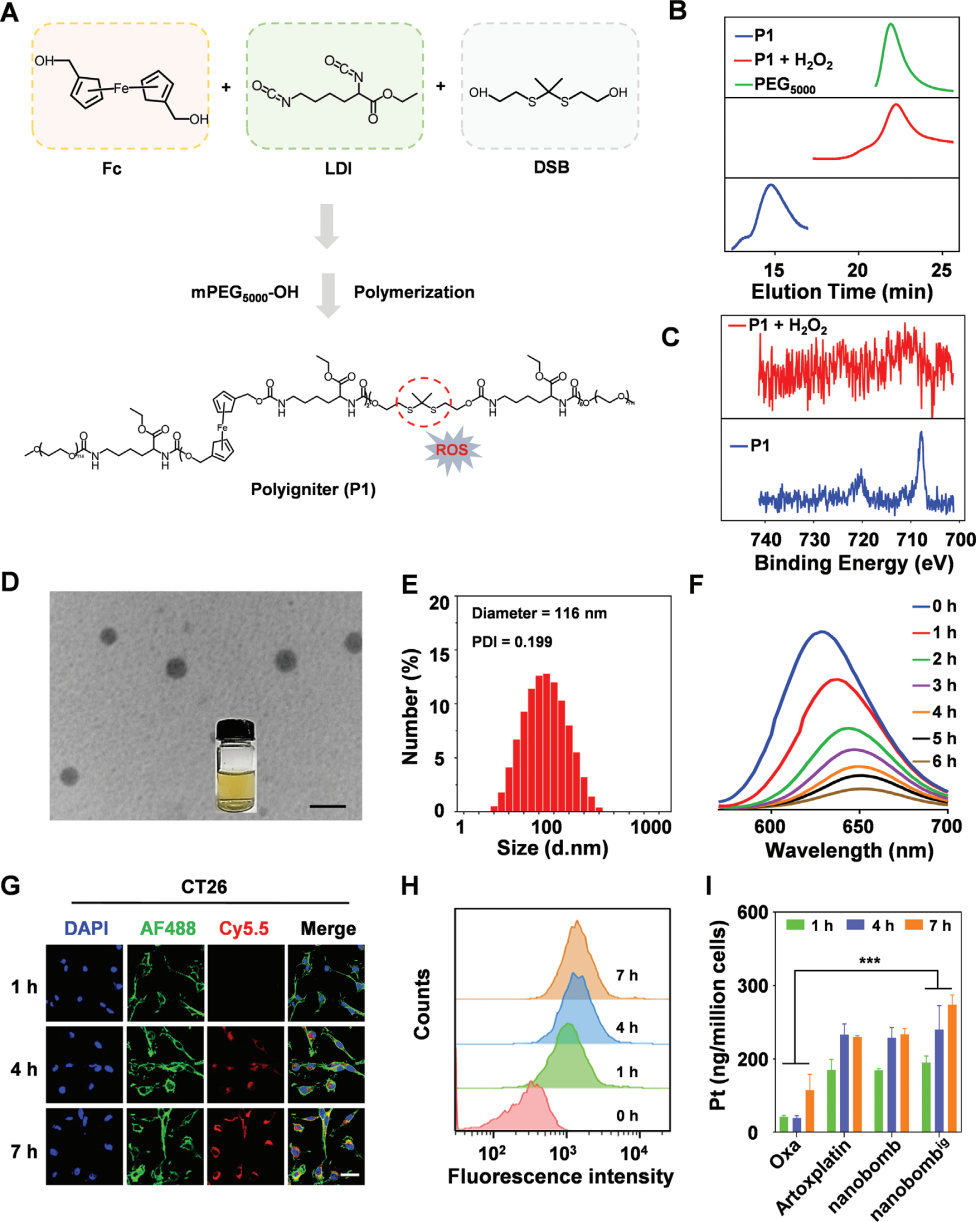
The nanobomb^ig^ could release Pt triggered by ROS, which could be internalized by cancer cells. A) Schematic illustration of the assembly of P1. B) Degradation of P1 after incubation with 10 mm H_2_O_2_ as monitored by GPC. C) Characterization of P1 before and after being treated with H_2_O_2_ (10 mm) via X‐ray photoelectron spectroscopy (XPS). D) Representative TEM image of nanobomb^ig^. Scale bar = 200 nm. E) Hydrodynamic diameters of nanobomb^ig^ by DLS. F) The change in fluorescence intensity curve of nanobomb^ig^ encapsulated with Nile red (*E*_x_ = 549 nm) after being treated with H_2_O_2_ (10 mm) for 6 h. G) Representative CLSM images of CT26 cells treated with Cy5.5‐labeled nanobomb^ig^ labeled by Cy5.5 at 1, 4, and 7 h, respectively. The blue fluorescence came from the cell nuclei stained with DAPI. The red and green fluorescence came from Cy5.5 and Alexa Fluor 488 Phalloidin, respectively. Scale bar = 20 µm. H) Intracellular uptake of nanobomb^ig^ loaded with Cy5.5 by FCM at 1, 4, and 7 h, respectively. I) ICP‐MS quantification of intracellular Pt uptake in CT26 cells treated with Oxa, Artoxplatin, nanobomb, and nanobomb^ig^ (2.5 µm Pt) at 1, 4, and 7 h, respectively. *** *p* < 0.001.

Fc is a stable hydrophobic organometallic complex containing Fe^2+^,^[^ ^20^ ^]^ which is considered as an ideal exogenous Fe^2+^. To supplement the cancer cells with exogenous Fe^2+^, a ROS‐sensitive polymeric polyigniter (P1) containing Fc was synthesized via a condensation polymerization of 1,1″‐Ferrocenedimethanol (Fc), 2,2″‐(propane‐2,2‐diylbis (sulfanediyl))bis(ethan‐1‐ol) (DSB), and L‐lysine diisocyanate (LDI), which was end‐caped with methoxyl‐polyethylene glycol (mPEG_5000_‐OH) (**Figure** **2**A). The weight‐average molecular weight (Mw), number‐average molecular weight (Mn), and polydispersity index (PDI) of the polyigniter were determined to be 28 363 Da, 14 590 Da, and 1.94, respectively, by gel permeation chromatography (GPC) (Table S1, Supporting Information). The degradation of P1 in the presence of 10 mm H_2_O_2_ was confirmed by GPC, suggesting the ROS‐responsiveness of P1. The result showed that the GPC trace of P1 was similar to mPEG_5000_‐OH after degradation, suggesting that P1 was possibly completely degraded into mPEG_5000_‐OH and other small pieces (Figure 2B). The ^1^H NMR characterization indicated that the molecular weight of the polyigniter was 14 590 Da, suggesting that there were two average units of Fc in each polyigniter chain (Figure S10, Supporting Information). Meanwhile, X‐ray photoelectron spectroscopy (XPS) was used to detect the binding energy of Fe in P1. The results demonstrated that the Fe peak of P1 had a binding energy of 707.8 eV (Figure 2C, blue line), while the Fe peak in P1 preincubated with H_2_O_2_ had a binding energy of 711.2 eV (Figure 2C, red line). The initial peaks of P1 disappeared after the addition of H_2_O_2_ and the newly generated peak had a binding energy that coincided with Fe^3+^, indicating that Fc in P1 was oxidized to a +3‐valence oxidation state.

Subsequently, polyigniter was used to encapsulate Artoxplatin to form the nanobomb^ig^ with a spherical morphology and a uniform diameter of ≈100 nm (Figure 2D). Moreover, the average hydrodynamic diameter of nanobomb^ig^ was further confirmed to be 116 nm by dynamic light scattering (DLS) with a polydispersity index (PDI) of 0.199 (Figure 2E). We also found that the nanobomb exhibited a uniform spherical shape with a homogeneous diameter of around 70 nm (Figure S11, Supporting Information). Artoxplatin encapsulation efficiency and loading capacity in nanobomb^ig^ were also evaluated. The results showed that Artoxplatin encapsulation efficiency was 45.9%, and the loading capacity was 4.59% (Figure S12A, Supporting Information). In addition, the release of Pt from nanobomb^ig^ was measured and the results indicated that there was a gradual increase in drug release at H_2_O_2_ 10 mm from 0 to 48 h. The drug release was 14.10% (PBS) and 77.66% (H_2_O_2_ = 10 mm) at 48 h (Figure S12B, Supporting Information). A stability test showed that nanobomb^ig^ remained stable for several days in 10% fetal bovine serum (FBS) and mouse serum (Figure S13, Supporting Information). To further study the kinetics of dual responsiveness of P1, the fluorescence spectra of nanobomb^ig^ encapsulated with Nile red were measured after the introduction of H_2_O_2_. The results showed that the fluorescence intensity of nanobomb^ig^ decreased over time in H_2_O_2_ pretreatment, with a half‐life of 2.56 h, indicating rapid dissociation of the nanoparticles (Figure 2F).

The internalization of nanomedicines by cancer cells is an important premise for their anticancer activity. To visualize the internalization of the nanobomb^ig^ by CT26 cells, a Cy5.5 fluorescent dye was used to label the nanobomb^ig^ for subsequent study by confocal laser scanning microscopy (CLSM) (Figure 2G) and FCM (Figure 2H). The results showed that a time‐dependent uptake of the nanobomb^ig^ by the cells was observed, since the red fluorescence at 7 h was more intense than at both 1 and 4 h, respectively (Figure 2G). A similar trend was also obtained by FCM (Figure 2H). As there are Pt and Fe elements in the nanobomb^ig^, it is feasible to quantify the Pt and Fe by inductively coupled plasma mass spectrometry (ICP‐MS). The results demonstrated that CT26 cells had an uptake of 173.90 ng of Pt/million cells after 7 h of nanobomb^ig^ incubation, which was ≈3.02 times that of Oxa at the same incubation time (Figure 2I). And the intracellular total iron was 705.73 ng per million cells after 7 h incubation with nanobomb^ig^, which was ≈1.96 times that of Oxa‐treated cells (Figure S14, Supporting Information). To further mimic the physiological conditions in vivo, 3D tumor spheroids based on CT26 cells were applied to verify the cellular internalization of the nanobomb^ig^. We found that the red fluorescence in the same depth portion of the 3D tumor spheres was gradually intensified from 12 to 24 h (Figure S15, Supporting Information), indicating efficient uptake of nanobomb^ig^ by the 3D multicellular tumor spheres. Overall, the nanobomb^ig^ could be efficiently internalized by cancer cells, and had the potential to release drugs in the tumor microenvironment.

### 2.4 In Vitro Cytotoxicity of the Nanobomb^ig^

**Figure 3:**
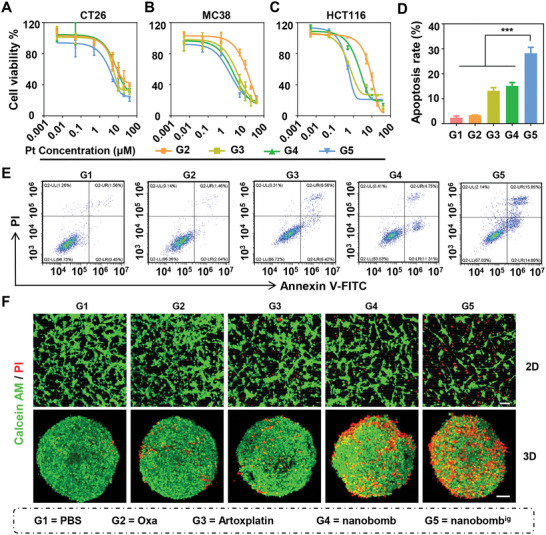
In vitro cytotoxicity of the nanobomb^ig^ on colorectal cancer cells and 3D spheroids. A‐C) Cell viability of CT26, MC38, and HCT116 cells treated with various drugs, including Oxa, Artoxplatin, nanobomb, and nanobomb^ig^ at 48 h. D) The apoptosis rates E) and representative FCM images of CT26 cells treated with various treatments, including Oxa, Artoxplatin, nanobomb, and nanobomb^ig^ at 48 h. F) Representative images of 2D cells and 3D cell spheroids based on CT26 cells stained with Calcein‐AM/PI with various treatments including Oxa, Artoxplatin, nanobomb, and nanobomb^ig^. Calcein‐AM‐labeled cells were live shown in green fluorescence, and PI‐labeled cells were dead shown in red fluorescence. Scale bar = 100 µm. *** *p* < 0.001.

Subsequently, three colorectal cancer cell lines were chosen, including CT26, MC38, and HCT116, to evaluate the in vitro antitumor activity of nanobomb^ig^. The results showed that nanobomb^ig^ had the strongest cytostatic effect in CT26 cells among all drugs tested with an IC_50_ value of 3.20 µm, whereas the IC_50_ value of Oxa was 5.30 µm (**Figure** **3**A). Moreover, the advanced potency of nanobomb^ig^ was also recapitulated in the other two cell lines tested (Figure 3B,C). Subsequently, an apoptosis assay was performed in CT26 cells and the results showed that the nanobomb^ig^ induced a significantly higher apoptosis rate (30.44%) than Oxa (15.16%) (Figure 3D,E). Furthermore, a live and dead cell staining assay was performed to investigate the cell‐killing effect of nanobomb^ig^ on 2D cells and 3D spheroids. The results revealed that the tumor cells and spheroids treated with PBS or Oxa displayed mainly green fluorescence (live cells), while those treated with nanobomb^ig^ showed great red fluorescence (dead cells) (Figure 3F). The above results together indicated that nanobomb^ig^ has a more efficient antitumor activity in vitro.

### 2.5 The Nanobomb^ig^ could Promote ROS Generation and Enhance ICD Effect

**Figure 4:**
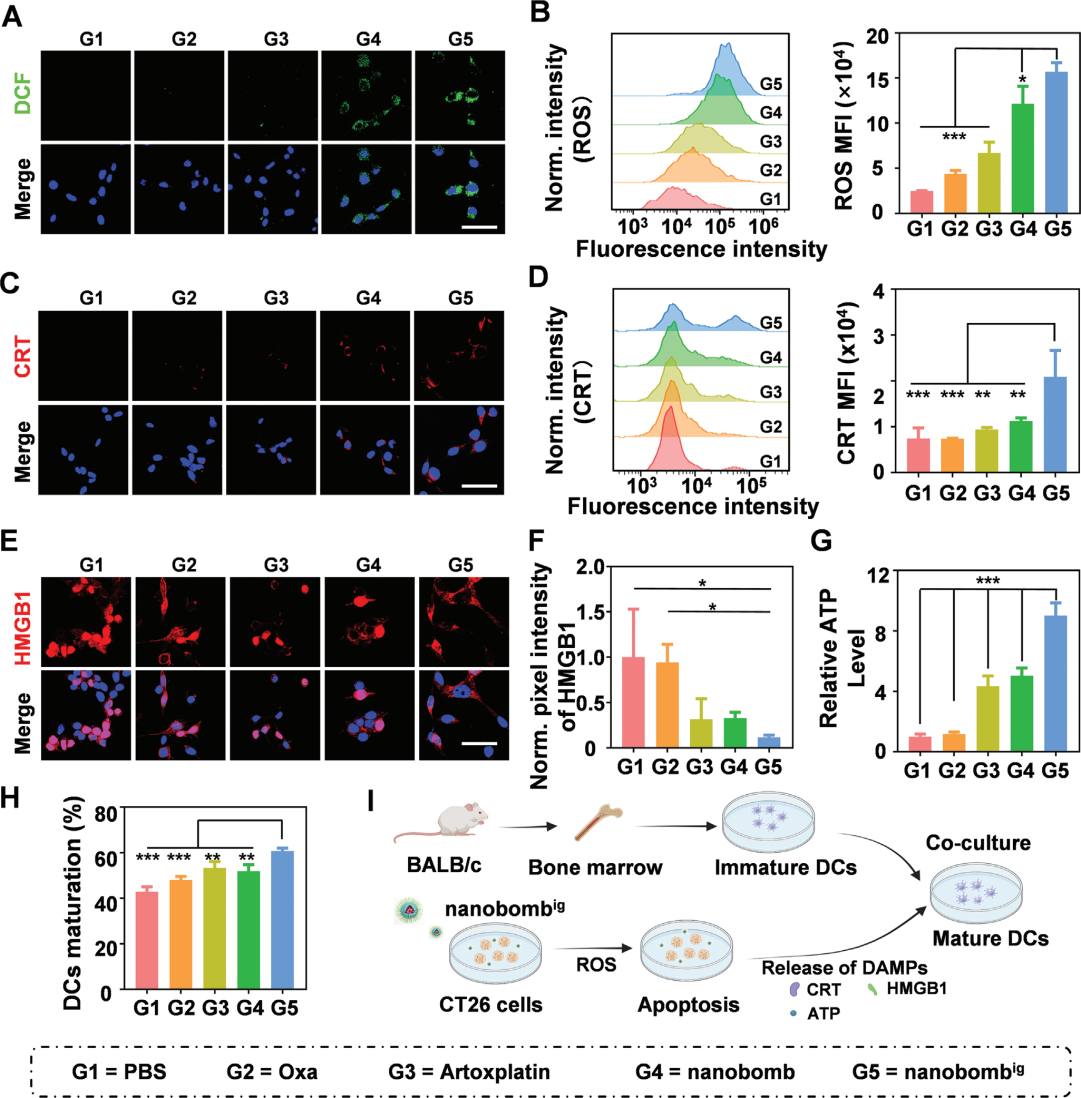
The nanobomb^ig^ induced more ROS generation, resulting in a stronger ICD effect and the maturation of BMDCs. A) The ROS detection by DCFH‐DA in CT26 cells via CLSM. B) Semiquantification of ROS generation via FCM. C) Representative CLSM images of the exposure of CRT in CT26 cells after various treatments including Oxa, Artoxplatin, nanobomb, and nanobomb^ig^. D) The exposure of CRT by FCM. E) Representative CLSM images of the release of HMGB1 CT26 cells after various treatments including Oxa, Artoxplatin, nanobomb, and nanobomb^ig^. F) Quantification of HMGB1 release in (E) by pixel intensity. G) Extracellular ATP levels in CT26 cells after various treatments including Oxa, Artoxplatin, nanobomb, and nanobomb^ig^. H) Semiquantitative study of BMDCs maturation cocultured with CT26 cells with various pretreatments by FCM. I) Schematic illustration of increased generation of ROS triggered by the nanobomb^ig^, resulting in a stronger ICD effect, and further promoting the maturation of BMDCs in vitro. Scale bar = 50 µm. *p < 0.05, **p < 0.01, ***p < 0.001.

We then assessed whether the Fc and ART released by the nanobomb^ig^ could promote intracellular ROS generation via a fluorescent probe of DCHF‐DA by CLSM and FCM. The results showed that CT26 cells treated with nanobomb^ig^ exhibited strong green fluorescence by CLSM (**Figure** **4**A). Further semiquantification by FCM indicated that the fluorescence intensity of CT26 cells treated with nanobomb^ig^ was nearly 1.3‐fold higher than that of cells treated with nanobomb, and almost 4 times that of the cells treated with Oxa (Figure 4B). These results demonstrated that the nanobomb with Fc units can significantly facilitate the intracellular ROS generation.

Next, we investigated whether the intracellular ROS upregulated by nanobomb^ig^ can induce an efficient ICD effect, stimulate the release of DAMPs, and promote DCs maturation. First, the exposure of CRT was detected by CLSM and FCM. On the one hand, the CLSM results indicated that CT26 cells treated with nanobomb^ig^ exhibited stronger red fluorescence (CRT) than those treated with other compounds (Figure 4C). On the other hand, the further semiquantitative FCM study confirmed that there was higher expression of CRT in cells treated with the nanobomb^ig^, resulting in 1.9 times and 2.8 times higher fluorescence intensity than that of cells treated with nanobomb and Oxa, respectively (Figure 4D). Second, the efflux of HMGB1 was investigated. The results showed that HMGB1 (red) was predominantly fused to the cell nucleus (blue) in CT26 cells treated with PBS, whereas CT26 cells treated with the nanobomb^ig^ elicited a dramatic reduction of nuclear HMGB1, indicating that the nanobomb^ig^ could facilitate more translocations of HMGB1 (Figure 4E,F). Third, there was the highest level of ATP in the supernatants of CT26 cells treated with the nanobomb^ig^, which was 1.8‐fold higher than that of cells treated with nanobomb, and almost 8 times higher than that of cells treated with Oxa (Figure 4G). Finally, CT26 cells with various treatments were subsequently cocultured with bone marrow dendritic cells (BMDCs) to investigate the maturation of BMDCs. The results showed that the proportion of mature BMDCs (CD80^+^CD86^+^) reached up to 60.7% in the nanobomb^ig^ treatment group, which was 1.5 times higher than those treated with PBS (42.9%) (Figure 4H; and Figure S16, Supporting Information). In summary, all these results revealed that the increased ROS triggered by the nanobomb^ig^ could induce stronger ICD effect and further promote the maturation of DCs (Figure 4I).

### 2.6 The Nanobomb^ig^ Regulates the Cellular Metabolism and Upregulates PD‐L1 Expression

**Figure 5:**
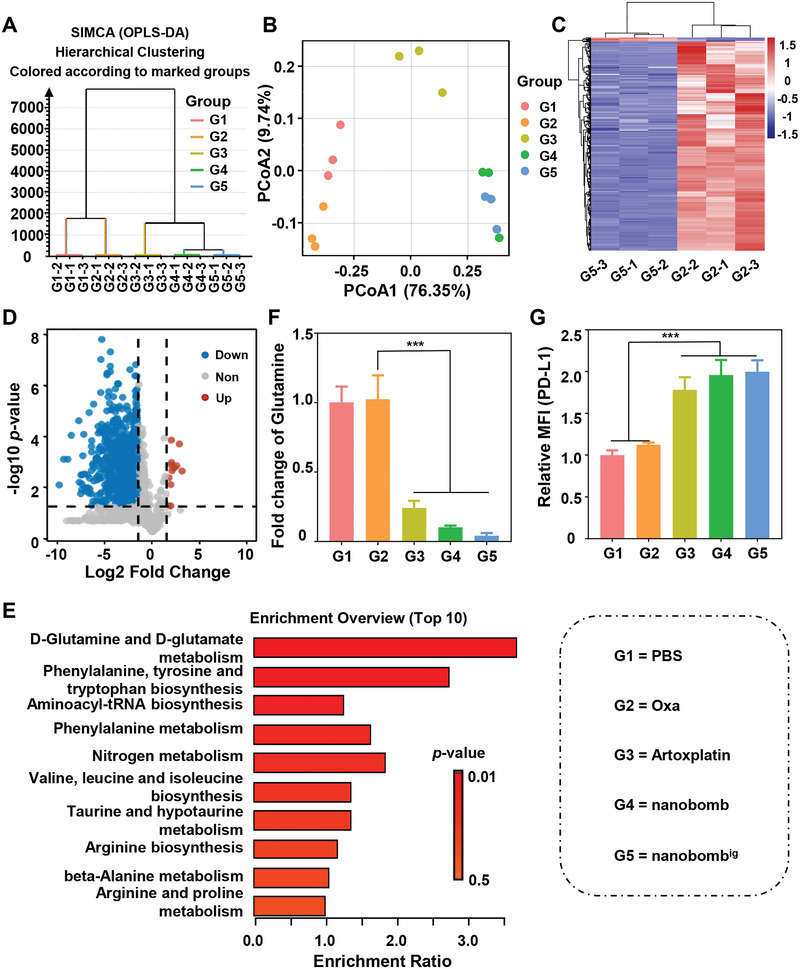
Metabolomic analysis unveils the nanobomb^ig^ down‐regulate the levels of intracellular glutamine, and up‐regulate the expression of PD‐L1 in cancer cells. A) Clustering dendrogram showing the clustering structure. B) PCoA analysis shows significant differences between different groups. C) Heatmap D) and Volcano plot based on the different metabolites between the cells treated with Oxa and the nanobomb^ig^. Different metabolites were recognized as fold change > 2, VIP > 1, and *p* < 0.05. E) Top ten KEGG pathway annotation of different metabolites in cells treated with Oxa and the nanobomb^ig^. F) Fold changes of glutamine in different groups. G) Expression levels of PD‐L1 in CT26 cells after various treatments. ***p < 0.001.

Given that ROS are key regulators of redox balance closely related to cellular metabolism, ^[^ ^21^ ^]^ we performed a metabolomic analysis based on an unbiased liquid chromatography/mass spectrometry (LC‐MC/MS) to further interrogate the effect of nanobomb^ig^ on cell metabolism. The hierarchical clustering analysis results over the 1266 quantified metabolites showed that the changes in metabolites were clearly distinguished among the various treatments (**Figure** **5**A). Further, the principal co‐ordinates analysis (PCoA) based on the 1266 quantified metabolites clearly separated the cells treated with the PBS, Oxa, Artoxplatin, nanobomb, and nanobomb^ig^, as 76.35% and 9.74% of all detectable metabolites were accounted for the principal component 1 and 2, respectively (Figure 5B). As shown in Figure 5C,D, there were 721 differential metabolites (fold change > 2, VIP > 1, and *p* < 0.05) in the nanobomb^ig^‐treated cells compared to that in Oxa, of which 706 and 15 were significantly increased and decreased, respectively. Furthermore, 68 differential metabolites, including 31 decreased and 37 increased, were found in the nanobombig‐treated cells compared to that in the nanobomb group (Figure S17A,B, Supporting Information). In particular, the Kyoto Encyclopedia of Genes and Genomes (KEGG) pathway analysis highlighted that the pathway of D‐Glutamine and D‐glutamate metabolism was significantly enriched in the nanobomb^ig^‐treated cells compared to that in Oxa (Figure 5E). Meanwhile, the KEGG analysis of differential metabolites between cells treated with nanobomb and nanobomb^ig^ also revealed significant enrichment of glutamine‐related metabolic pathways (Figure S17C, Supporting Information). Considering the important role of glutamine in glutathione (GSH) synthesis,^[^ ^22^ ^]^ we then interrogated whether nanobomb^ig^ could decrease the generation of GSH in CT26 cells. The results showed that the nanobomb^ig^‐treated cells exhibited more than a 6.0‐fold decrease in the GSH/GSSG ratio measured in the Oxa‐treated cells (Figure S18, Supporting Information). Meanwhile, significant changes in GSH and the related metabolites were also found in nanobomb^ig^‐treated cells compared with Oxa‐treated cells (Figure 5F; and Figure S19, Supporting Information). Some reported works indicated that decreased GSH/GSSG ratio is associated with increased ROS production and enhanced endoplasmic reticulum stress in tumor cells, which can promote enhanced ICD effects and immunogenicity, ultimately facilitating the activation of antitumor immunity.^[^ ^7^, ^13^, ^23^ ^]^

It has been reported that PD‐L1, a classical immune checkpoint molecule with the capacity to influence the clinical response of anti‐PD‐1/PD‐L1 therapy, is upregulated by glutamine limitation.^[^ ^22^ ^]^ To determine whether treatment of the nanobomb^ig^ could increase the expression of PD‐L1 in cancer cells, we performed FCM analysis to compare the expression levels of PD‐L1 in cells under various treatments. As shown in Figure 5G, the nanobomb^ig^‐treated cells exhibited a more than 2.0‐fold upregulation of the expression level of PD‐L1 measured in PBS‐treated cells (Figure 5G; and Figure S20, Supporting Information). Together, these observations supported that nanobomb^ig^ could downregulate the level of glutamine in cancer cells, which would increase PD‐L1 expression, and potentially enhance the efficacy of PD‐1/PD‐L1 blockade therapy simultaneously.

### 2.7 Biodistribution and Antitumor Efficacy of Nanobomb^ig^ In Vivo

Biosafety is the crucial prerequisite for nanomedicine to elicit the in vivo activity.^[^ ^24^ ^]^ First, hemolysis assay was conducted to evaluate the biocompatibility of nanobomb^ig^, and no hemolysis of the blood was noticed (Figure S21, Supporting Information). Subsequently, biosafety evaluation of nanobomb^ig^ was studied on healthy KM mice. Oxa, Artoxplatin, nanobomb, and nanobomb^ig^ (3 mg kg^−1^ Pt) were i.v. injected into the mice every 3 days for a total of 4 times. The body weight of mice in each group were recorded every 3 days after the first injection. And the serum and major organs were collected 48 h after the last dose of drugs for physiological and biochemical study. The results showed that there was no significant change in body weight among various treatments (Figure S22, Supporting Information). Moreover, the biochemical indexes were also relatively normal in mice treated with the nanobomb^ig^ (Figure S23, Supporting Information). Additionally, no apparent pathological abnormality was found in the hematoxylin and eosin (H&E) staining images of the major organs in mice with different treatments (Figure S24, Supporting Information). In summary, the aforementioned results indicated the excellent biocompatibility of the nanobomb^ig^.

**Figure 6:**
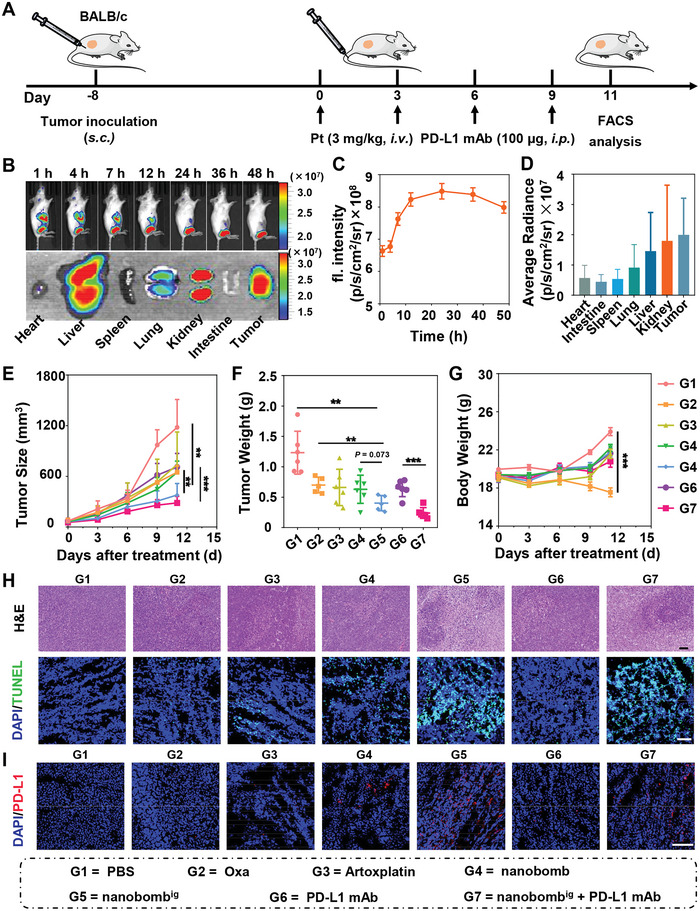
A combination of the nanobomb^ig^ with PD‐L1 mAb inhibited the tumor growth on a CT26 tumor model. A) Schematic illustration of the workflow for drug dosage. B) Biodistribution of the nanobomb^ig^ labeled with Cy7.5 by live fluorescence imaging on CT26 bearing mice after *i.v*. injection at different time points (top panel) and ex‐vivo imaging (bottom panel). C) Semiquantification of fluorescence intensity in tumor of mice at different time points D) and the ex vivo imaging of fluorescence intensity in major organs and the tumor at 48 h after i.v. injection. E) Tumor growth inhibition curves (*n* = 5–6). F) Tumor weight after 12 days treatment. G) The body weight change curves of mice. H) H&E staining (top panel), TUNEL assay (bottom panel) in tumors from each treatment group. I) Expression of PD‐L1 by immunofluorescence staining in tumors of mice with various treatments. Scale bar = 100 µm. **p < 0.01, ***p < 0.001.

We then explored the biodistribution of the nanobomb^ig^ on a subcutaneous CT26 tumor‐bearing mouse model (**Figure** **6**A). First, the nanobomb^ig^ loaded with Cy7.5 dye was prepared, and the fluorescence intensity at the tumor site was monitored after i.v. injection by an in vivo image system (IVIS Spectrum). The living image results showed that obvious fluorescence was detected in the tumor area at 1 h postinjection. Moreover, the fluorescence intensity gradually increased with time, reaching up to a peak at 24 h postinjection (Figure 6B (Top panel), C). Interestingly, the fluorescence signal persisted more than 48 h postinjection, indicating the excellent tumor retention of the nanobomb^ig^. To quantify the biodistribution of the nanobomb^ig^, the main organs and tumors were collected at 48 h postinjection. The ex vivo imaging showed that the liver and kidney were the main organs for the accumulation of nanobomb^ig^ (Figure 6B (Bottom panel), D). Further semiquantification of the fluorescence at the tumor site indicated the fluorescence continued to increase in the first 24 h (Figure 6B). Moreover, the average fluorescence intensity in the tumors ranked first among all tissues, which was significantly higher than that in the lung, heart, spleen, and intestine (Figure 6D).

Subsequently, the in vivo antitumor activity of the nanobomb^ig^ and the synergistic benefit with PD‐L1 mAb were investigated in CT26 tumor‐bearing female BALB/c mice (Figure 6A). The results showed similar and limited tumor inhibitory effect of Oxa, Artoxplatin, and nanobomb in tumor‐bearing mice. However, a vigorous tumor growth inhibitory effect was found in the mice treated with nanobomb^ig^ (Figure 6E,F). Specifically, the average tumor volume and weight in mice treated with nanobomb^ig^ were both only 1/3 of those in mice treated with PBS. More importantly, the mice treated with Oxa experienced obvious body weight loss by nearly 10%, while there was no evident change for the mice treated with nanobomb^ig^ till the end of the study (Figure 6G), indicating the excellent biosafety of nanobomb^ig^.

The aforementioned metabolomic study revealed that the nanobomb^ig^ could facilitate the expression of PD‐L1 on tumor cells, laying the foundation for the combination of PD‐L1 mAb and the nanobomb^ig^ for tumor immunotherapy. The results showed that nanobomb^ig^ plus PD‐L1 mAb resulted in significant inhibition of tumor growth rate compared to PD‐L1 mAb alone (Figure 6E,F). Specifically, the average tumor volume treated with the combination of nanobomb^ig^ and PD‐L1 mAb was only 285.3 mm^3^, which was roughly 2/5 and 1/4 in mice treated with PD‐L1 mAb monotherapy or PBS, respectively. Taken together, the above findings indicated that the combination of nanobomb^ig^ and PD‐L1 mAb could result in more robust antitumor efficacy.

Furthermore, cell death in the tumors was evaluated by H&E staining and the terminal deoxynucleotidyl transferase‐mediated deoxyuridine triphosphate‐nick end labeling (TUNEL) assay. First, the H&E staining indicated that there was extensive nuclear shrinkage and disappearance in the tumor tissues treated with nanobomb^ig^ alone and in combination with PD‐L1 mAb (Figure 6H (Top panel)). Second, the TUNEL assay of frozen slices revealed increased green fluorescence indicative of DNA damage in tumors of mice receiving nanobomb^ig^ (Figure 6H (Bottom panel)). Third, consistent with the above findings, the expression of PD‐L1 was upregulated in tumor tissues of mice treated with nanobomb^ig^ (Figure 6I).

### 2.8 Antitumor Immune Response of Nanobomb^ig^ In Vivo

**Figure 7:**
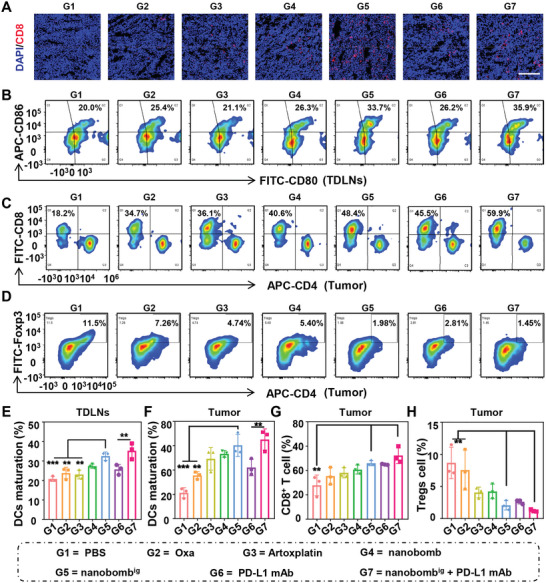
The nanobomb^ig^ could stimulate robust systemic anti‐tumor immunity, which can be combined with PD‐L1 mAb for synergistic chemotherapy and immunotherapy. A) CD8^+^ T cells in tumors of mice with various treatments by immunofluorescence staining. Scale bar = 200 µm. B) The CD80^+^CD86^+^ DCs on CD11c^+^ cells gate within TDLNs by FCM. C) The CD8^+^ and CD4^+^ T cells and D) Tregs (CD4^+^Foxp3^+^) on CD3^+^ T cells gate within tumor tissues by FCM. E) The percentages of mature DCs (CD80^+^CD86^+^) populations within TDLNs. F) The percentages of mature DCs, G) CD8^+^ T cells, and H) Tregs populations within tumor tissues *n* = 3, **p < 0.01, ***p < 0.001.

To further evaluate the immune response stimulated by nanobomb^ig^, the infiltration of CD8^+^ T cells in tumor tissues was analyzed in mice with various treatments by immunofluorescence staining. The results showed that there was intensive red fluorescence (CD8^+^ T cells) in tumor tissues of mice treated with nanobomb^ig^ (**Figure** **7**A), suggesting there would be higher proportion of tumor‐infiltrating activated cytotoxic T cells.

To examine the antitumor immunity induced by the nanobomb^ig^ alone and in combination with PD‐L1 mAb, the spleen, tumor tissues, and tumor‐draining lymph nodes (TDLNs) of mice with various treatments were harvested at the end of the tumor suppression study. The results illustrated that the proportion of mature DCs (CD11c^+^CD80^+^CD86^+^) in TDLNs of mice treated with nanobomb^ig^ reached 32.5%, which was almost 1.4 times that of Oxa (23.83%) (Figure 7B,E). Moreover, a similar trend was observed in tumor tissues. The ratio of mature DCs in the tumors of mice treated with nanobomb^ig^ was ≈1.7 times that of Oxa (60.4% vs 35.6%) (Figure 7F; and Figure S25, Supporting Information). Nevertheless, compared with nanobomb^ig^ alone, nanobomb^ig^ plus PD‐L1 mAb did not further facilitate DCs maturation in TDLNs and tumors, but both were higher than that of PD‐L1 mAb monotherapy. The above results indicated the nanobomb^ig^ could result in the maturation of DCs in TDLNs and tumor tissues. To verify whether mature DCs could further activate the response of T cells, we then evaluated the activation of CD8^+^ T cells, which are effective killer cells in antitumor immunity. The results showed that compared with the mice treated with PBS and Oxa, nanobomb^ig^ could dramatically increase the proportion of cytotoxic T lymphocytes (CD3^+^CD8^+^) in TDLNs (Figure S26, Supporting Information), spleens (Figure S27, Supporting Information), and tumors (Figure 7C,G), but not CD4^+^ T lymphocytes. The percentage of CD3^+^CD8^+^ T cells at the tumor site in mice treated with nanobomb^ig^ alone was 46.0%, which was 1.3 times and 1.6 times that of Oxa and PBS, respectively (Figure 7C,G). Further, numerous studies indicated that the major immunosuppressive cells, usually known as regulatory T cells (Tregs, CD4^+^Foxp3^+^) and myeloid‐derived suppressor cells (MDSCs, CD45^+^CD11b^+^Gr‐1^+^), could significantly inhibit the activation of antitumor immunity.^[^ ^25^ ^]^ Subsequently, we found the infiltration of Tregs (Figure 7D,H) and MDSCs (Figure S28, Supporting Information) at the tumor site decreased remarkably in the mice treated with nanobomb^ig^, which were 4.1‐fold and 2.5‐fold lower than that in mice treated with PBS. Similar results were observed in mice treated with nanobomb^ig^ + PD‐L1 mAb. Taken together, all the above results indicated that nanobomb^ig^ could induce the activation of antitumor immunity in CT26‐bearing mice by facilitating DCs maturation and remodeling the tumor immune microenvironment. These effects would result in the synergistic effect of chemotherapy and immunotherapy, and ultimately enhance the tumor treatment effect.

## 3 Conclusions

Cancer immunotherapy has been actively studied in recent years and has yielded tremendous clinical benefits for several malignancies. However, poor immunogenicity seriously hampers the clinical application of anticancer immunotherapy. The enhanced cellular immunogenicity triggered by ICD accompanied with the release of DAMPs, would subsequently facilitate DCs maturation, and ultimately elicit robust T‐cell immunity. Thus, there is an urgent need to develop a nanoparticle strategy that boosts stronger ICD effect to improve the efficacy of tumor immunotherapy.

In this work, we reported a cancer nanobomb^ig^ composed of (Pt(IV)) prodrug (Artoxplatin) and oxidatively cleavable polyigniter containing Fc units, for ROS generation in situ to enhance the ICD effect, facilitating the maturation of DCs and the activation of T cell immune response. We demonstrated that nanobomb^ig^ could effectively accumulate at the tumor site in CT26 tumor‐bearing mouse model. Moreover, the nanobomb^ig^ could release Oxa, ART, and Fc, and then generate highly toxic ROS through the catalytic reaction of ART and Fc, enhancing cell toxicity and the ICD effect and ultimately evoking a robust antitumor immunity. More importantly, the metabolomic analysis revealed that the nanobomb^ig^ significantly affected the metabolisms of glutamine‐related metabolic pathways, thereby decreasing the level of GSH in cancer cells. We found for the first time that upregulated PD‐L1 on cancer cells treated with nanobomb^ig^ potentially improved the antitumor efficacy when combined with PD‐L1 mAb. In our animal studies, we unraveled the synergistic effect of nanobomb^ig^ and PD‐L1 mAb treatment, reflected by a significant decrease in tumor volume compared with that in the PBS control and PD‐L1 mAb alone.

Given the low response rate to cancer immunotherapy, our findings proposed a promising strategy to enhance cancer chemo‐immunotherapy and further potential in combination with immune checkpoint blockade, providing promise in clinical applications and translational prospects.

## Conflict of Interest

The authors declare no conflict of interest.

## Supporting information

Supporting Information

## Data Availability

The data that support the findings of this study are available from the corresponding author upon reasonable request.
