# Supplementary material for: Cancer Nanobombs Delivering Artoxplatin with a Polyigniter Bearing Hydrophobic Ferrocene Units Upregulate PD‐L1 Expression and Stimulate Stronger Anticancer Immunity
Source: Adv Sci (Weinh). 2023 May 11;11(4):2300806. doi: 10.1002/advs.202300806 (PMC10811492; doi:10.1002/advs.202300806)
Supplement: Supplementary file 1 — Supporting Information [file ADVS-11-2300806-s001.pdf]

ADVANCED SCIENCE
Open Access

Supporting Information

for *Adv. Sci.*, DOI 10.1002/advs.202300806

Cancer Nanobombs Delivering Artoxplatin with a Polyigniter Bearing Hydrophobic Ferrocene Units Upregulate PD-L1 Expression and Stimulate Stronger Anticancer Immunity

Yongchao Gao, Hanchen Zhang, Lin Tang, Feifei Li, Li Yang, Haihua Xiao, Johannes Karges, Weihua Huang, Wei Zhang and Chaoyong Liu**

ADVANCED SCIENCE
Open Access

Supporting Information

for *Adv. Sci.*, DOI 10.1002/advs.202300806

Cancer Nanobombs Delivering Artoxplatin with a Polyigniter Bearing Hydrophobic Ferrocene Units Upregulate PD-L1 Expression and Stimulate Stronger Anticancer Immunity

Yongchao Gao, Hanchen Zhang, Lin Tang, Feifei Li, Li Yang, Haihua Xiao, Johannes Karges, Weihua Huang, Wei Zhang and Chaoyong Liu**

Supporting Information

Cancer Nanobombs Delivering Artoxplatin with a Polyigniter Bearing Hydrophobic Ferrocene Units Upregulate PD-L1 expression and Stimulate Stronger Anticancer Immunity

Yongchao Gao[1,2,3,4], *Hanchen Zhang*[5,6], *Lin Tang*[7], *Feifei Li*[7], *Li Yang*[8], *Haihua Xiao*[5,6], *Johannes Karges*[9], *Weihua Huang*[1,2,3,4], *Wei Zhang*[1,2,3,4,10,11] *, *and Chaoyong Liu*[7] *

1. Department of Clinical Pharmacology, Xiangya Hospital, Central South University, 87 Xiangya Road, Changsha 410008, PR China.
2. Institute of Clinical Pharmacology, Central South University, Hunan Key Laboratory of Pharmacogenetics, 110 Xiangya Road, Changsha 410078, PR China.
3. Engineering Research Center of Applied Technology of Pharmacogenomics, Ministry of Education, 110 Xiangya Road, Changsha 410078, PR China.
4. National Clinical Research Center for Geriatric Disorders, 87 Xiangya Road, Changsha 410008 Hunan, PR China.
5. Beijing National Laboratory for Molecular Sciences, Key Laboratory of Polymer Physics and Chemistry and CAS Key Laboratories of Organic Solids, Institute of Chemistry, Chinese Academy of Sciences, Beijing, 100190, P. R. China.
6. University of Chinese Academy of Sciences, Beijing 100049, P. R. China.
7. Beijing Advanced Innovation Center for Soft Matter Science and Engineering, College of Life Science and Technology, Beijing University of Chemical Technology, Beijing, 100029 P. R. China.
8. Institute of Chinese Medical Sciences, State Key Laboratory of Quality Research in Chinese Medicine, University of Macau, 999078, Macao.
9. Faculty of Chemistry and Biochemistry, Ruhr-University Bochum, Universitätsstrasse 150, 44780 Bochum, Germany.
10. Hunan Provincial Tumor Hospital and the Affiliated Tumor Hospital of Xiangya Medical School, Central South University, Changsha, 410006, P. R. China.
11. Key Specialty of Clinical Pharmacy, The First Affiliated Hospital of Guangdong

Pharmaceutical University, Guangzhou 510080, P. R. China.

TABLE OF CONTENTS

Supplementary materials and methods **1-12**
Figure S1. .... **13**
Figure S2. .... **14**
Figure S3. .... **15**
Figure S4. .... **16**
Figure S5. .... **17**
Figure S6. .... **18**
Figure S7. .... **19**
Figure S8 .... **20**
Figure S9. .... **21**
Figure S10. .... **22**
Figure S11 .... **23**
Figure S12 .... **24**
Figure S13. .... **25**
Figure S14. .... **26**
Figure S15. .... **27**
Figure S16. .... **28**
Figure S17. .... **29**
Figure S18. .... **30**
Figure S19. .... **31**
Figure S20. .... **32**
Figure S21 .... **33**
Figure S22. .... **34**
Figure S23. .... **35**
Figure S24. .... **36**
Figure S25. .... **37**
Figure S26. .... **38**
Figure S27. .... **39**
Figure S28. .... **40**
Figure S29. .... **41**
Figure S30. .... **42**
Figure S31. .... **43**
Table S1. .... **44**
References **45**

Supplementary materials and methods

Materials

Mercaptoacetic acid, trifluoroacetic acid, acetone, lithium aluminium, succinic anhydride, 1,1'-Ferrocenedimethanol, (2,2'-(propane-2,2-diylbis(sulfanediyl))bis (ehan-1-ol)) and 3-(4,5-dimethylthiazol-2-yl)-2,5-diphenyltetrazolium bromide (MTT) were purchased from Aladdin (Shanghai, China) and Energy Chemical. L-lysine diisocyanate were bought from Energy Chemical (Shanghai, China). Oxaliplatin (Oxa) was purchased from Shandong Boyuan Chemical Company, Shandong, China. Artesunate (ART) was purchased from Hvsf United Chemical Materials Company (Beijing, China). RPMI-1640 medium, Dulbecco's Modified Eagle Medium (DMEM) with 4.5 g glucose, penicillin/streptomycin (P/S), 0.25% trypsin-EDTA, and Fetal Bovine Serum (FBS) were purchased from Gibco (Gran Island, NY, U.S.A.).

General instruments

Dynamic light scattering (DLS) was conducted by Malvern Zetasizer Nano ZS90 (Malvern Instruments, Malvern, UK). Inductively Coupled Plasma (ICP) analysis was conducted using Inductively Coupled Plasma Optical Emission Spectrometer (Agilent technologies 7700 series, U.S.A.). The morphology and size were measured by transmission electron microscope (TEM, Hitachi HT 7700, Japan). Flow cytometry (FCM) was conducted by Cytomics FC500 Flow Cytometry (Beckman Coulter, U.S.A.). Confocal laser scanning microscopy (CLSM) was accomplished by ZEISS LSM880. ^{1}H NMR spectra and ^{13}C NMR were measured by a 400 or 300 MHz NMR spectrometer (Bruker, USA) at room temperature. High resolution mass spectrometry (HRMS) was conducted by Agilent 1290 UPLC/6540 Q-TOF. All OD values were recorded by SpectraMax M3. X-ray photoelectron spectroscopy (XPS) was performed on the Thermo Scientific ESCALab 250Xi. Gel permeation chromatography (GPC) was conducted Agilent technologies LC-20A.

Cell lines and animals

All cells were purchased from the American Type Culture Collection (ATCC,

Manassas, VA, USA) cultured in RPMI-1640 medium, DMEM supplemented with 10% FBS (Gibco) (v/v), respectively, and incubated at 37°C in a 5% CO_2 atmosphere. Female BALB/c mice and KM mice (6 weeks) were obtained from Hunan SJA Laboratory (Hunan, China) and raised in SPF animal rooms. All animals were free access to standard diet and water, and kept indoors under 12 h day/12 h night. All animal work was done in accordance with the National Institutes of Health's Guide for the Use and Care of Laboratory Animals and was approved by the Institutional Animal Care and Use Committee (IACUC) of Central South University. (2018sydw0258).

Synthesis of Artoxplatin

The compound Oxa(IV)-OH was synthesized according to a previously reported protocol.[1] Briefly, Oxa (3.0 g, 7.6 mmol) was added to 50-mL round-bottomed flask with 20 mL 30% H_2O_2. The reaction solution was centrifugated after 24 h reaction at room temperature. After washing with water (5 mL) and cold ether (5 mL) for 2 times, the solid was dried under vacuum to obtain Oxa(IV)-OH as white powder (2.8 g, yield 86%).

ART (2.3 g, 6.0 mmol) was dissolved in 20 mL of anhydrous ether with the addition of dicyclohexylcarbodiimide (618 mg, 3.0 mmol). The mixture solution was stirred at room temperature overnight, and filtered to obtain clear solution. The solvent was evaporated and the residue was dissolved in 20 mL DMF with the addition of Oxa(IV)-OH (429 mg, 1.0 mmol). The mixture was heated and stirred at 50 °C overnight to obtain a clear yellow solution. The solvent was evaporated and purified using thin-layer chromatography to obtain Artoxplatin (382 mg, 33%).

Synthesis of Polymer 1 (P1)

L-lysine diisocyanate (260 mg, 1.15 mmol) was quickly added to an anhydrous DMF solution (5 mL) of 1,1'-Ferrocenedimethanol (47.6 mg, 0.2 mmol) and ROS sensitive linker (2,2'-(propane-2,2-diylbis(sulfanediyl))bis(ehan-1-ol)) (157 mg, 0.8 mmol). After magnetic stirring for another 12 h at room temperature, $mPEG_{5000}$-OH (1 g, 0.2 mmol) was added to the reaction mixture. After magnetic stirring for another 24 h at

50 °C, the mixture was added into 10 mL of deionized water under sonication, followed by dialysis in a dialysis bag (MWCO: 8000-14000 Da). After 72 h, the solution was freeze-dried under reduced pressure to give P1 (800 mg) as a yellow powder.

Degradation of P1 after H_2O_2 treatment by GPC

P1 (2 mg/mL, 2 mL) was added to a 5 mL centrifugation tube containing H_2O_2 (10 mM, 2 mL), then the mixture was incubated at 37 °C for 5 h. GPC test was carried out after the above solution was dialyzed and lyophilized.

XPS examination of P1

P1 (1 mg/mL, 1 mL) was challenged with H_2O_2 (10 mM， 1 mL) at 37 °C for 24 h. The solution was freeze-dried under reduced pressure. Lyophilized samples were then tested by XPS.

Nile red encapsulation and release

Nile Red dye was used as a model compound to visually examine the effect of oxidation on the dye release from the nanobomb^{ig}. Artoxplatin (2 mg), P1 (20 mg) were co-dissolved in 1 mL DMSO was mixed with 200 μL of Nile Red solution (1 mg/mL in DMSO) and stirred for 2 h. The mixture was added to 9 mL water drop by drop. The mixture was then transferred to a dialysis bag (MWCO: 3500 Da) and dialyzed for 24 h. After that, the solution of Nile Red loaded nanoparticle was collected and diluted to 20 mL. The effect of the H_2O_2-induced oxidation on the Nile Red release was studied by mixing 3 mL of Nile Red loaded nanoparticle solution with H_2O_2 (10 mM) and incubating at room temperature. The fluorescence spectra of the solutions were recorded at different time intervals (SpectraMax M3, λ_{ex} = 540 nm) to monitor the release of Nile Red dye from the nanobomb^{ig}.

Preparation and characterization of nanobomb^{ig}

Artoxplatin (5 mg), P1 (50 mg) were co-dissolved in 1 mL DMSO. Then, under the condition of stirring, the solution was quickly injected into 10 mL water to self-assemble to form nanoparticles (nanobomb^{ig}). Free drugs and organic solvent were

removed through dialysis against deionized water for 24 h. The concentration of Pt in nanobomb^{ig} was assessed *via* ICP-MS (Agilent technologies 7700 series, U.S.A.). The hydrodynamic size nanobomb^{ig} was detected *via* a DLS device (Malvern Zetasizer Nano, UK). The morphology and shape of nanobomb^{ig} were visualized with a TEM device (Hitachi, Japan).

Nanoparticles uptake in the cells by CLSM and FCM

A cover slide was placed in the bottom of each well of a 24-well plate. CT26 cells (1×10^5) in 1 mL media were added to each well and incubated at 37 °C for 12 h. Then the cells were treated with nanobomb^{ig}@Cy5.5 (2.5 μM Pt) for 1 h, 4 h, or 7 h, respectively. After being washed with cold PBS, the cells were fixed with paraformaldehyde. Cell nuclei were stained with DAPI (ThermoFisher Scientific). The cytoskeleton was stained with Actin (Beyotime). Subsequently, images were collected with CLSM. (DAPI, λ_{ex} = 405 nm, λ_{em} = 460 nm, Cy5.5, λ_{ex} = 673 nm, λ_{em} = 692 nm, Actin: λ_{ex}= 496 nm, λ_{em}= 516 nm)

To perform flow cytometry, CT26 cells were seeded on 12-well plates at 2×10^5 cells per well and incubated at 37 °C for 12 h. Cells were then treated with nanobomb^{ig}@Cy5.5 (2.5 μM Pt) for 1 h, 4 h, or 7 h, respectively. Finally, the cells were harvested to examine the intracellular uptake by FCM.

The uptake and apoptosis test on 3D tumor spheroids

1% agarose gel solution (50 μL) was added to each 96-well plate. 1,500 CT26 cells (200 μL) were added to each well. On the 7th day, the cell spheres were formed. For analysis of cellular uptake of nanoparticles, the 3D spheroids were treated with nanobomb^{ig}@Cy5.5 (5 μM Pt) for 12 h and 24 h. After being washed with cold PBS, the spheroids' uptake of nanoparticles was measured *via* CLSM. Images were captured at intervals of 5 μm from top to bottom of the live spheroids. (nanobomb^{ig}@Cy5.5: λ_{ex}= 673 nm, λ_{em}= 692 nm)

For the apoptosis test, the cells and spheroids were treated with PBS, Oxa, Artoxplatin, nanobomb, and nanobomb^{ig} for 24 h, respectively (5 μM Pt). After being washed with cold PBS, the cells and spheroids were stained with Calcein AM/PI Cell

Viability Kit (KeyGEN BioTECH). Subsequently, images were collected with CLSM. (Calcein-AM: λ_{ex} = 495 nm, λ_{em} = 515 nm, propidium iodide (PI): λ_{ex}= 493 nm, λ_{em}= 617 nm)

Pt release kinetics of nanobomb^{ig}

The in vitro drug release kinetics study of nanoparticles was carried out by dialysis using PBS or an aqueous solution (H_2O_2 = 10 mM) as the release medium. Seal 5 mL of nanobomb^{ig} at a Pt concentration of 100 μM in a dialysis bag (molecular retention of 3500), and then immerse them in 200 mL of release medium in a beaker covered with aluminum foil. Keep the beaker at 37°C while shaking at 100 rpm. At various time points, 1 mL of sample solution was taken from the dialysate and measured by ICP-MS. The platinum released from the micelles was expressed as the percentage of cumulative platinum in the dialysate to the total platinum in nanobomb^{ig}.

MTT assay of various Pt containing drugs on various cancer cells

CT26, MC38, and HCT1116 cells were seeded in 96-well plates (4×10^3 cells/well) and incubated at 37 °C for 12 h. Subsequently, the cells were treated with PBS, Oxa, Oxa+2ART, Artoxplatin, nanobomb, and nanobomb^{ig} at various Pt concentrations ranging from 0.005 μM to 40 μM of Pt for 48 h. The Art concentration ranged from 0.01 μM to 80 μM in Oxa+2ART group. To verify the effect of Fe^{2+}, the cells were pre-treated with 10 μM of ferrous sulfate. After incubation for 10 hours, the cells were washed three times with cold PBS and then followed the above experimental procedure. Cells were then incubated with 10% MTT (5 mg/mL solution in PBS buffer) and the plates were further allowed to incubate with cells for another 4 h. Acidified SDS solution was then added (100 μL) was added in 96-well plates for each well, and the plates were kept in the dark for an additional 12 h. Measurements of absorbance were subsequently made with a Bio-Rad plate reader (SpectraMax M3) at 570 nm (peak absorbance) and subtracted at 650 nm (background absorbance).

Intracellular ROS assessments

CT26 cells were seeded on 24 wells plate with cell slides covered and 12 wells plate at a density of 1×10^5 and 2×10^5 respectively. Cells were treated with Oxa, Artoxplatin,

nanobomb, and nanobomb^{ig} at the same concentration of Pt (25 μM) for 7 h. Subsequently, the culture medium was replaced with a serum-free medium and then incubated with ROS indicator DCFH-DA (10 μM) for 30 mins. The samples were detected by CLSM and flow cytometry, respectively.

To verify the effect of Fe^{2+}, the cells were pre-treated with 10 μM of ferrous sulfate. After incubation for 10 h, the cells were washed three times with cold PBS and then followed the above experimental procedure.

Apoptosis analysis

Cellular apoptosis was assessed with an Annexin V-FITC apoptosis detection kit (Elabscience) according to the manufacturer's instructions. In brief, CT26 cells were seeded on 12-well plates at 2×10^5 cells per well. After 12 h incubation, cells were treated with PBS, Oxa, Oxa+2ART, Artoxplatin, nanobomb, and nanobomb^{ig}, respectively (Pt at 5 μM in Figure 1, and at 2.5 μM in Figure 3) for 48 h. The cells were then washed with PBS, and incubated with Annexin/PI reagent in the dark for 15 min at 37 °C. Thereafter, the cells were immediately measured with FCM.

For cells requiring iron pretreatment, medium contain 10 μM ferrous sulfate was added to each well and discarded after 10 h of incubation. Then the cells were washed three times with cold PBS, and followed the drug treatment.

Live/dead stain of cancer cells

CT26 cells were seeded on 6-well plates at a density of 3×10^5 cells per well. After 12 h incubation, cells were treated with PBS, Oxa, Artoxplatin, nanobomb, and nanobomb^{ig}, respectively (2.5 μM Pt) for 48 h. After this time, the media was removed and the cells were then washed with cold PBS for three times. The cells were further incubated with Calcein AM/PI Cell Viability Kit (KeyGEN BioTECH) for 15 min, and the cell survival/death was assessed by CLSM. (Calcein-AM: λ_{ex} = 495 nm, λ_{em} = 515 nm, PI: λ_{ex}= 493 nm, λ_{em}= 617 nm)

Measurement of cell surface CRT

CRT exposure was evaluated by FCM and CLSM. For FCM analysis, CT26 cells

were seeded on 12-well plates at 2×10^5 cells per well. After 12 h incubation, cells were treated with PBS, Oxa, Oxa+2ART, Artoxplatin, nanobomb, and nanobomb^{ig}, respectively (Pt at 25 μM) for 6 h. Cells were then collected and blocked with 1% BSA (Beyotime), and then further incubated with CRT primary antibody (ab211962, Abcam) at 4°C for 1 h. After washing with PBS for 3 times, cells were incubated with the Alexa Fluor 488-conjugated secondary antibody (ab150077, Abcam) for 30 min and then the surface fluorescence was assayed with FCM. For CLSM analysis, cover slides were placed in the bottom of each well of a 24-well plate, and CT26 cells (1×10^5) in 1 mL complete media were added to each well and incubated at 37 °C for 12 h. Then the cells were then treated with PBS, Oxa, Artoxplatin, nanobomb, and nanobomb^{ig} at an equal Pt concentration for 6 h. Next, the cells were washed with PBS and fixed in 4% paraformaldehyde solution for 20 min, followed by incubation with 1% BSA (Beyotime) for 30 min. Then the cells were incubated with primary CRT antibody at 4 °C overnight, and then incubated with the Alexa Fluor 555-conjugated secondary antibody (ab150078, Abcam) after three washes with PBS. Nuclei were counterstained with DAPI (ThermoFisher Scientific) and observed under CLSM using 405 nm and 555 nm lasers for visualizing nuclei and CRT exposure on the cell membrane, respectively. (Alexa Fluor 488: λ_{ex} = 495 nm, λ_{em} = 519 nm, Alexa Fluor 555: λ_{ex} = 555 nm, λ_{em} = 580 nm)

Measurement of the release of HMGB1

The passively released HMGB1 was measured *via* CLSM analysis. In brief, a cover slide was placed in the bottom of each well of a 24-well plate, and CT26 cells (1×10^5) in 1 mL media were added to each well and incubated at 37 °C for 12 h. Cells were then treated with different formulations like the CRT for 24 h (10 μM Pt), and washed with PBS for three times. Then, the cells were fixed in 4% paraformaldehyde for 20 min and permeabilized with 0.1% Triton X-100 for 10 min. Thereafter, cells were blocked with 1% BSA for 30 min, and then incubated with HMGB1 primary antibody (ab216986, Abcam) overnight at 4 °C. After washing with PBS for 3 times, cells were incubated with Alexa Fluor 555-conjugated antibody for 30 min. Thereafter, cell nuclei were stained with DAPI, and imaged with a confocal microscope. (Alexa Fluor

555: λ_{ex} = 555 nm, λ_{em} = 580 nm)

ATP release assay

The amount of ATP in the medium was measured with an ATP assay kit (Beyotime) according to the manufacturer's instructions. CT26 cells were seeded in 96-well plates at a density of 4×10^3 cells per well and cultured for 24 h. Subsequently, the cells were treated with PBS, Oxa, Oxa+2ART, Artoxplatin, nanobomb, and nanobomb^{ig} at an equal Pt concentration (5 μM Pt) for 24 h. Afterward, the culture medium was collected and the concentration of ATP was evaluated according to the manufacturer's instructions. The luminescence of the samples was measured by a microplate reader (SpectraMax M3).

BMDCs maturation *in vitro*

Bone-marrow derived dendritic cells (BMDCs) were obtained and cultured according to previously reported methods.[2] Briefly, BMDCs were collected from 6 weeks-old female BALB/c mice and cultured in RPMI-1640 medium supplement with 10% FBS, granulocyte-macrophage colony-stimulating factor (GM-CSF) (20 ng/mL, Beyond), and interleukin-4 (IL-4) (10 ng/mL, Beyond) at 37 °C with 5% (v/v) CO2. After 5 days of culture, CT26 cells pretreated with different drugs were co-cultured with BMDCs for 24 h. After co-incubation, cells were stained with the corresponding antibody (anti-CD11c-PE, anti-CD80-FITC, anti-CD86-APC, Biolegend, USA) for BMDCs mature *via* FCM.

Metabolomics analysis

Cells cultured in dishs (triplicates) were subjected to different treatments (PBS, Oxa, Artoxplatin, nanobomb, and nanobomb^{ig} (2.5 μM Pt)) for 48 h, which were next washed with cold PBS twice before being extracted with 500 μL ice-cold extraction solvent (100:180:120 ultrapure water: methanol: chloroform). After shaking well for 1 min, 150 μL ultrapure water was then added into the Eppendorf tube and centrifuged at 1000× *g* for 15 min at 4°C. 400 μL supernatant was transferred to liquid chromatography vials, spun dry, and reconstituted to 40 μL for metabolomic analysis. All metabolites were detected by UPLC (Ultimate 3000, ThermoFisher Scientific, San

Jose, CA, USA)-ESI-Qrbitrap-MS (Orbitrap Fusion Lumos, ThermoFisher Scientific, San Jose, CA, USA). Identification and relative quantification of the data were conducted by Compound discoverer (3.1). The normalized data was imported into the SIMCA-P version 14.1 to create an OPLS-DA model. The follow-up enrichment analysis of metabolomic was based on MetaboAnalyst 5.0 (https://www.metaboanalyst.ca/), and the significance of enriched pathways was calculated using Fisher's exact method.

Measurement of PD-L1 level in cancer cells

In order to determine the expression level of PD-L1, CT26 cells were cultured and treated with different formulations (2.5 μM Pt) like the HMGB1 for 48 h. Cells were stained with PE-PD-L1 (Biolegend, USA) for 1 h. Stained cells were analyzed by FCM.

Hemolytic Activity Study

For the hemolytic activity study, blood samples were obtained from mice and centrifuged at 358× *g*, 4°C for 5 min. The supernatant was discarded and replaced with PBS. This step was repeated three times. The following PBS addition led to a 1/5 dilution of red blood cells (RBCs). RBCs were incubated with Oxa, Artoxplatin, nanobomb, and nanobombig at a Pt concentration of 40 μM for 3 h at 37°C (with a RBCs:drug ratio 1:1). Sterile ddH2O was used as the positive control, while PBS was used as a negative control. After incubation, the intact erythrocytes were separated by centrifugation at 2240× *g*, 4°C for 5 min. The supernatants were transferred into 96-well plate, and the absorption at 541 nm was measured with a microplate reader. The hemolysis ratio was calculated using the following formula:

$$HP\ (\%) = (Dt-Dnc) / (Dpc-Dnc) \times 100\%$$

Dt-experimental group, Dnc-negative control group, Dpc-positive control group

***In vivo* toxicological evaluation**

The female KM mice (6 weeks) were randomly grouped (n=3 mice per group). Oxa, Artoxplatin, nanobomb, and nanobomb^{ig} (3 mg/kg Pt) were injected every three days

intravenously (*i.v.*) for a total of 4 times. The mice were monitored and weighed every three days after the first injection. Then, mice were sacrificed at 11 days after administration. Physiological and biochemical of mice blood samples were collected and examined.

Establishment of tumor model and biodistribution of nanobomb^{ig} *in vivo*

CT26 cells (1×10^6 in 100 μL) were injected subcutaneously (*s.c.*) in the right lateral dorsal side of each mouse. CT26 tumor-bearing mice were *i.v.* injection of nanobomb^{ig}@Cy7.5 (3 mg/kg Pt) when the tumor volume reached about 200 mm^3, followed by imaging with an IVIS Spectrum (PerkinElmer) at 1 h, 4 h, 7 h, 12 h, 24 h, 36 h and 48 h post-injection, respectively (Cy7.5, λ_{ex}: 745 nm, λ_{em}: 840 nm). 48 h post-injection, mice were sacrificed to collect tumors and major organs for *ex vivo* imaging. The fluorescence intensity (photos s^{-1} cm^{-2} sr^{-1}) was quantitatively analyzed by living image software.

Tumor models and treatment experiments

When there were ~100 mm^3 palpable tumors, mice were randomly divided into seven groups (n = 6) including PBS group, Oxa group, Artoxplatin group, nanobomb group, nanobomb^{ig} group, PD-L1 mAb group, and nanobomb^{ig}+PD-L1 mAb group. For groups containing Pt, mice were *i.v.* injection of the corresponding drugs (3 mg/kg Pt) on days 0, 3, 6, 9, respectively. And PD-L1 mAb (100 μg) was intraperitoneally (*i.p.*) administered to mice every 3 days for a total of 4 times. The tumor volume and mouse weight of each group was measured every three days, and the tumor volume (mm^3) was calculated from measurements by using the following formula: $L\times W^2/2$, where L represents the large diameter of the tumor, and W represents the small diameter of the tumor. The mice were sacrificed 48 h after the last treatment, tumor derived lymph nodes (TDLNs), tumor nodules and spleens were harvested for flow cytometry analysis.

Histopathological analysis

The solid tumors were harvested from tumor-bearing mice on the 11th day of drug injection for histological observation by standard hematoxylin and eosin (H&E)

staining and immunofluorescence staining. For H&E staining, the excised tumors and organs were fixed in 4% paraformaldehyde solution, embedded in paraffin, sectioned, and stained with H&E. The sections were then observed under a fluorescence microscope (IX83, Olympus). Twelve-μm thick frozen tissue sections were prepared for TdT mediated-dUTP Nick-End Labeling (TUNEL) assay and immunofluorescence staining. TUNEL assay was performed according to the instrument (Solarbio). For detecting the expression of PD-L1 and infiltration of $CD8^+$ T cells in tumor tissues, frozen tumor sections were fixed, and blocked with 1% BSA. Primary antibodies targeting PD-L1 (WLH2821, Wanleibio) and CD8 (MA1-84018, Invitrogen) were incubated overnight at 4℃ in the blocking solution and the following day for 30 min at room temperature. After extensive washing in PBS, the secondary antibody including anti-mouse Alexa Fluor 555-conjugate (ab150078, Abcam) was added to the blocking solution and incubated for 2 h. Nuclei were counterstained with DAPI (ThermoFisher Scientific). And then Images were detected and captured by CLSM.

Flow Cytometry analyses

Single-cell suspensions were prepared from TDLNs, tumors and spleens by mechanical dissociation, and followed by the treatment of red blood cell lysing buffer (Solabio) to remove red blood cells. 70-μm cell strainer was used to remove debris. All samples were washed and resuspended in PBS. After that, samples were blocked with 0.1% BSA in PBS and incubated with relevant antibodies for 50 min at room temperature. For characterizing T cells in TDLNs, tumors and spleens, cells were stained by anti-mouse CD3-PE, anti-mouse CD4-APC, anti-mouse CD8-FITC or anti-mouse Foxp3-FITC (Biolegend, USA). For analyzing DCs in tumor and lymph nodes, cells were stained by anti-mouse CD11c-PE, anti-mouse CD80-FITC, and anti-mouse CD86-APC (Biolegend, USA). For characterizing myeloid-derived suppressor cells (MDSCs) in tumor, cells were stained by anti-mouse CD45-Percp, anti-mouse CD11b-APC, and anti-mouse Gr-1-FITC (Biolegend, USA). For staining of intracellular antigens, cells were permeabilized using Foxp3 Fixation and Permeabilization Kit (eBioscience) before staining. Flow cytometric data acquisition was performed with CytExpert software, and analyzed by FlowJo software (TreeStar;

Ashland, OR). The gating strategies were shown in **Figure S29-31**.

Data analysis and statistics

All statistical analyses were performed either with Graph Pad Prism 8 (GraphPad Software, Inc., California) or the statistical environment R 3.5.2 (https://www.r-project.org/). All the data were presented as the mean ± SD. Statistical evaluations between two groups were carried out using unpaired two-tailed Student's t test, and for comparison between multiple groups, one-way ANOVA was used. Differences were considered statistical significantly when *P* values less than 0.05.

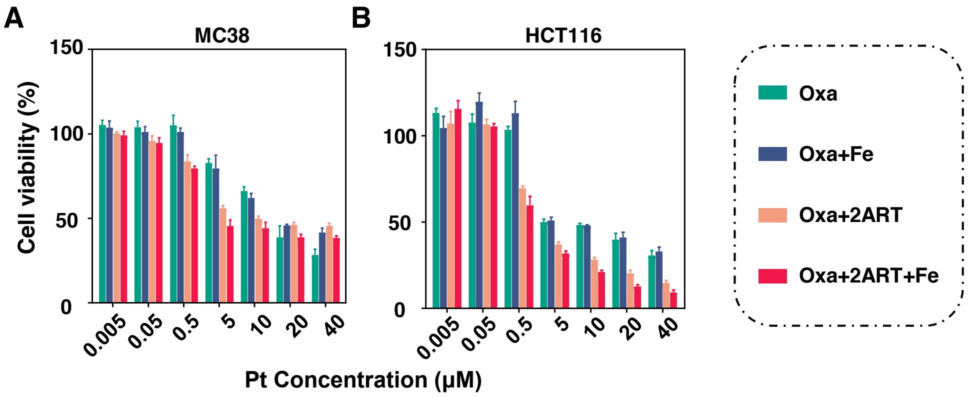

Figure S1. Cell viabilities of (A) MC38 and (B) HCT116 cells at 48 h after various treatment including Oxa and Oxa+2ART pretreatment with or not Fe^{2+}.

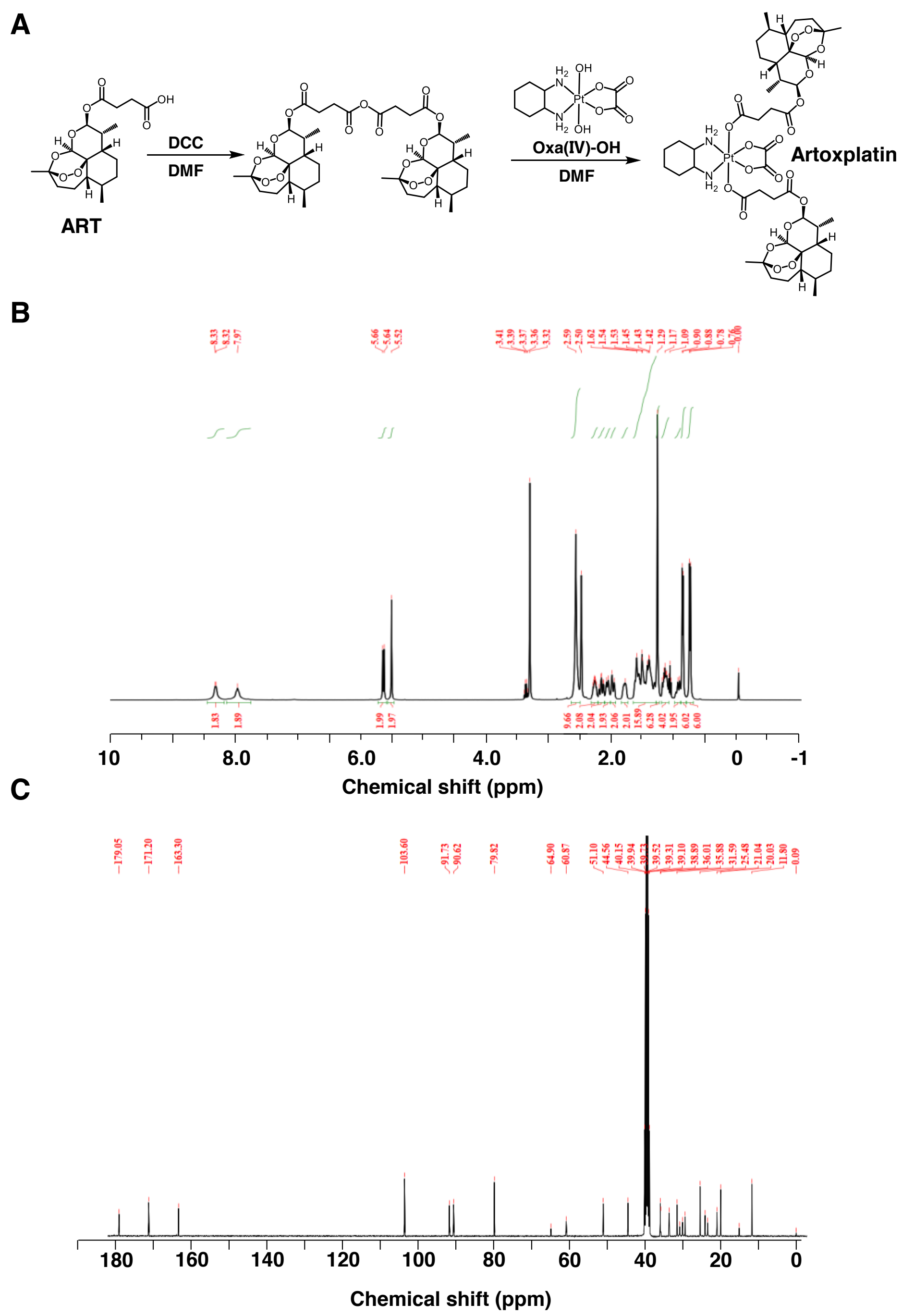

Figure S2. (A) Synthesis route of Artoxplatin. (B) ^{1}H NMR and (C) ^{13}C NMR spectra of Artoxplatin in $CDCl_3$ at 298 K.

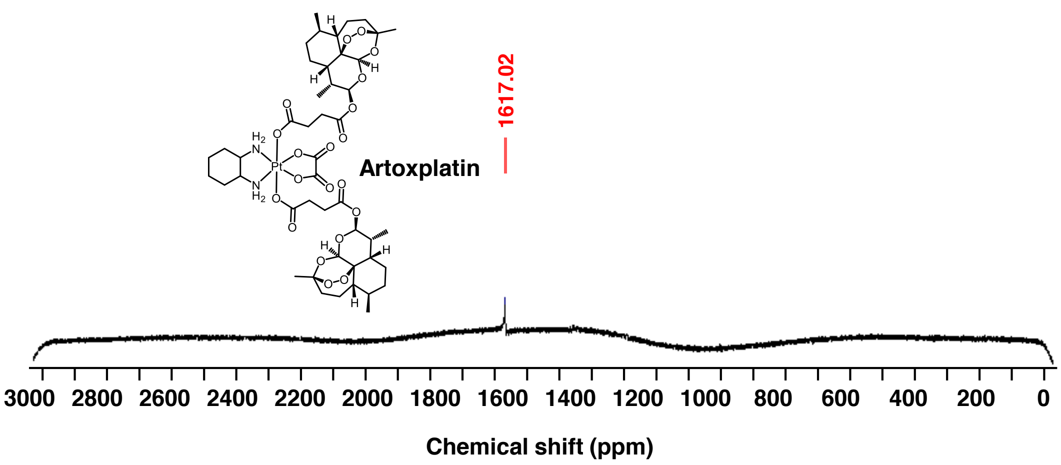

Figure S3. The ^{195}Pt NMR spectra of Artoxplatin in $CDCl_3$ at 298 K.

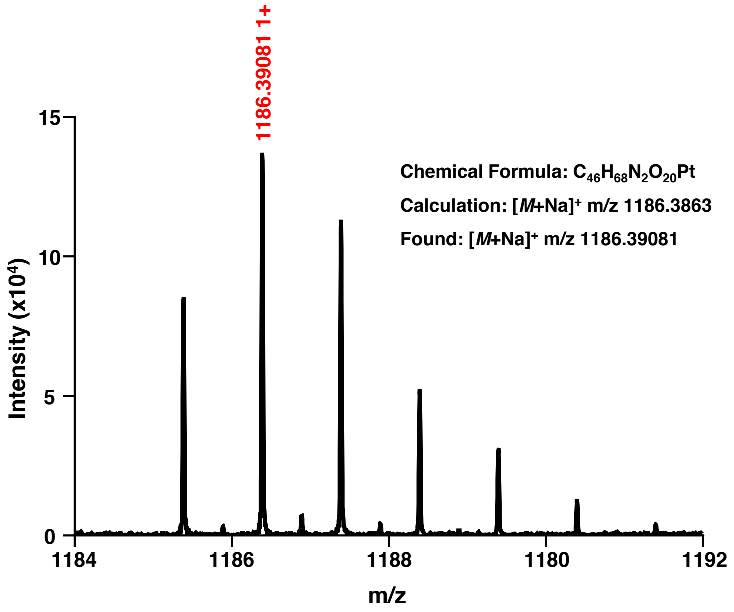

Figure S4. HR-ESI-MS spectra of Artoxplatin in CH_3OH.

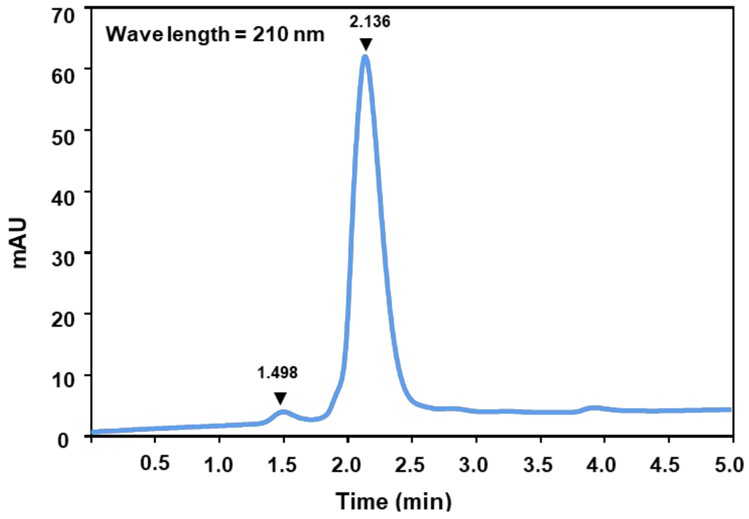

RT (min)	Width (min)	Area	Height	Area%
1.498	0.53	21.44	1.76	2.16
2.136	0.99	971.07	59.08	97.84

Figure S5. Chromatogram of Artoxplatin.

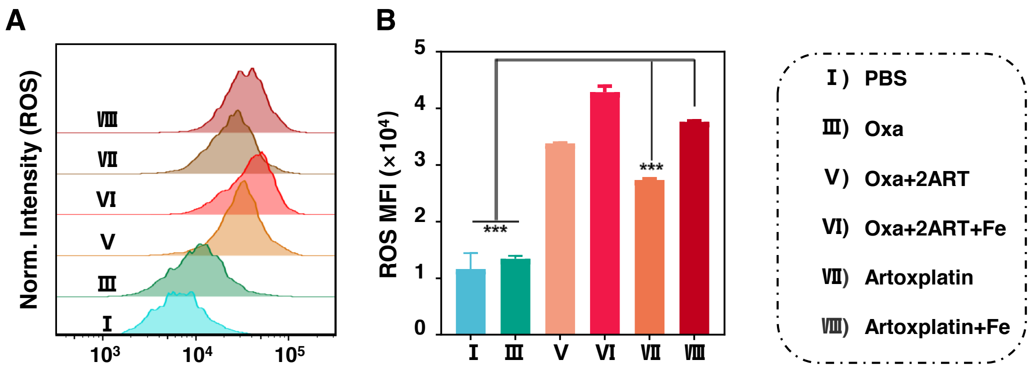

Figure S6. (A) Flow cytometric curves (B) and the corresponding quantification of ROS generation in CT26 cells treated with diverse Pt-containing drugs including Oxa, Oxa+2ART and Artoxplatin pretreatment with or not Fe^{2+}. ***$p < 0.001$.

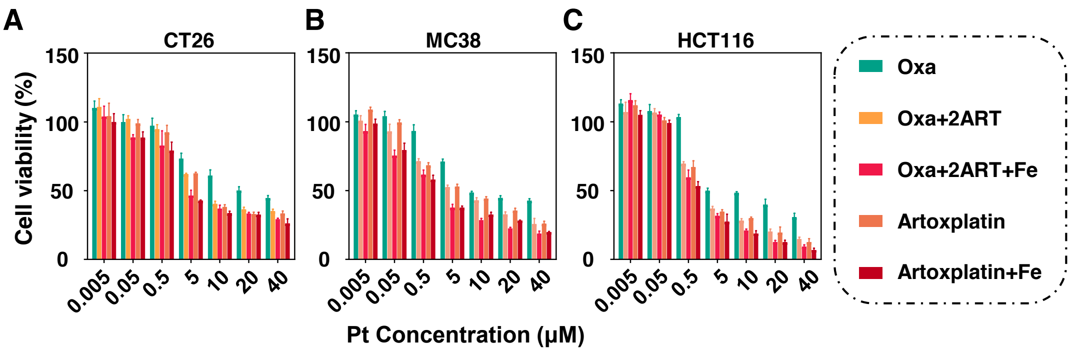

Figure S7. Cell viability of (A)CT26, (B) MC38, and (C) HCT116 cells with various treatments including Oxa, Oxa+2ART and Artoxplatin pretreatment with or not Fe^{2+} for 48 h.

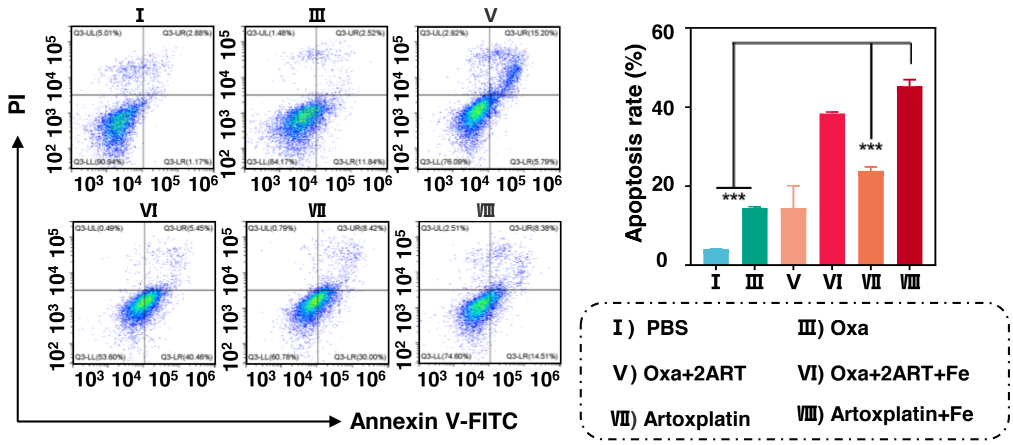

Figure S8. Apoptotic ratio and the representative FCM images in CT26 cells treated with various Pt-containing drugs (5 μM Pt) including Oxa, Oxa+2ART and Artoxplatin pretreatment with or not Fe^{2+} for 48 h. n = 3 per group. ***$p < 0.001$.

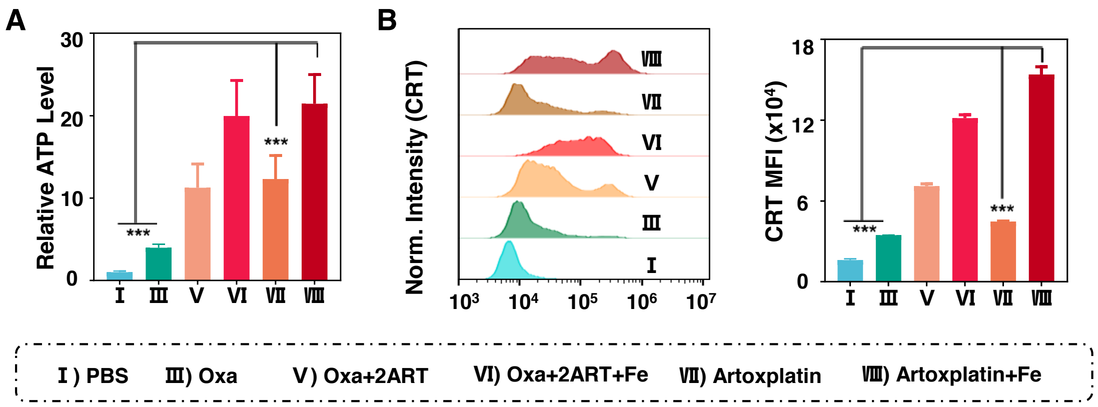

Figure S9. (A) Relative ATP level, and flow cytometry analyses and the corresponding quantification of CRT (B) in CT26 cells treated with various Pt-containing drugs including Oxa, Oxa+2ART and Artoxplatin pretreatment with or not Fe^{2+}. n = 3 per group. ***$p < 0.001$.

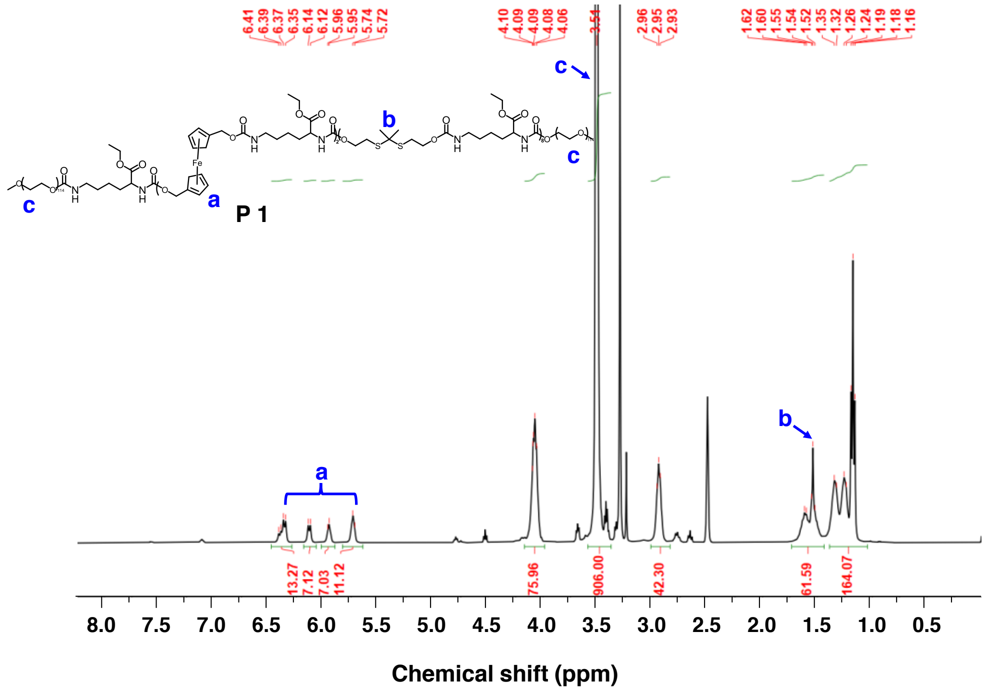

Figure S10. ^1H NMR spectra of Polyigniter (P1) in $CDCl_3$ at 298 K.

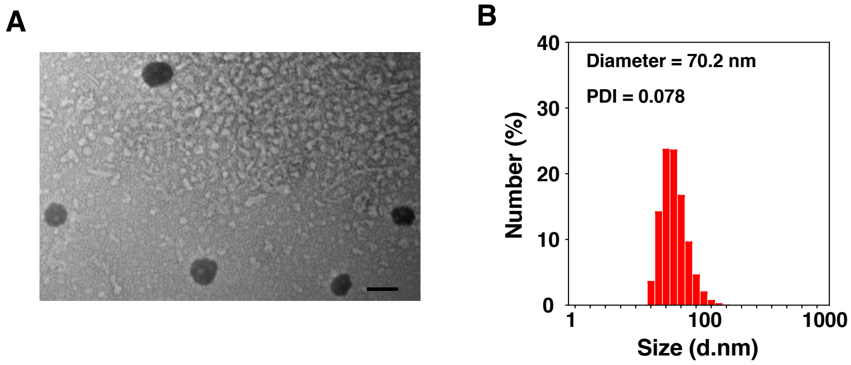

Figure S11. (A) Representative TEM image of nanobomb. Scale bar = 100 nm. (B) Hydrodynamic diameters of nanobomb by DLS.

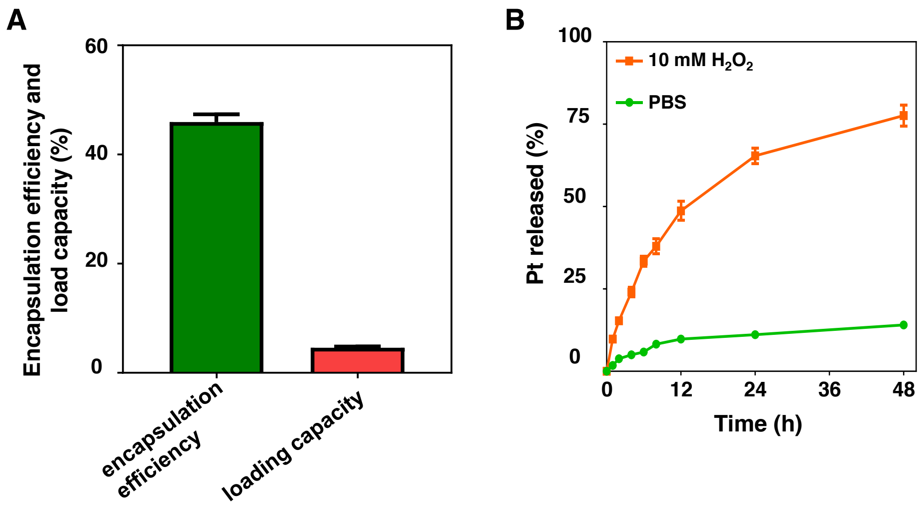

Figure S12. Artoxplatin encapsulation efficiency and load capacity of nanobomb[ig].

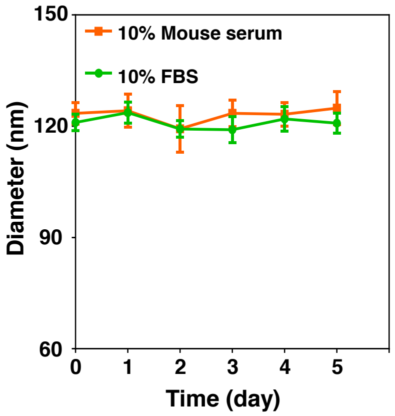

Figure S13. The stability (average diameter) of nanobomb^{ig} in 10% fetal bovine serum (FBS) and mouse serum within 6 d monitored by DLS.

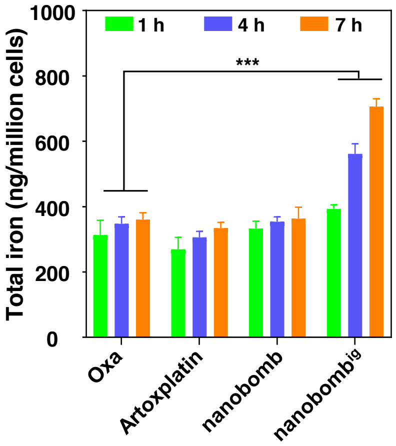

Figure S14. ICP-MS quantification of intracellular total iron concentration in CT26 cells treated with Oxa, Artoxplatin, nanobomb and nanobomb^{ig} (2.5 μM Pt) at 1h, 4 h and 7 h, respectively. ***$p < 0.001$.

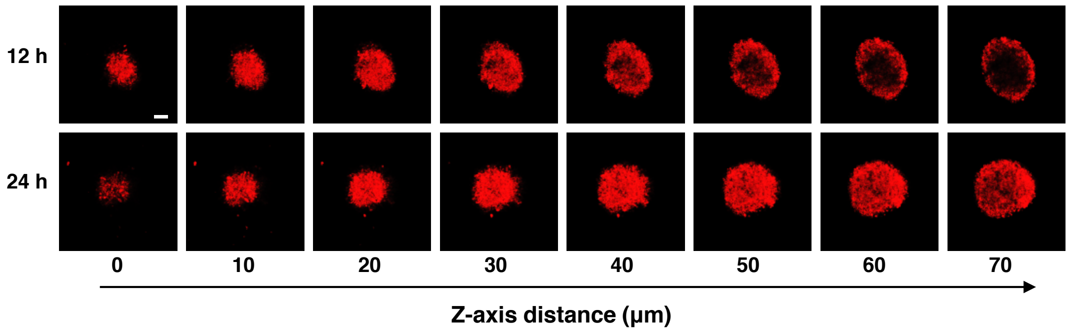

Figure S15. Representative CLSM images of nanobomb^{ig} labeled with Cy5.5 internalized by 3D tumor spheroids of CT26 cells at different incubation times. Scale bar = 100 μm.

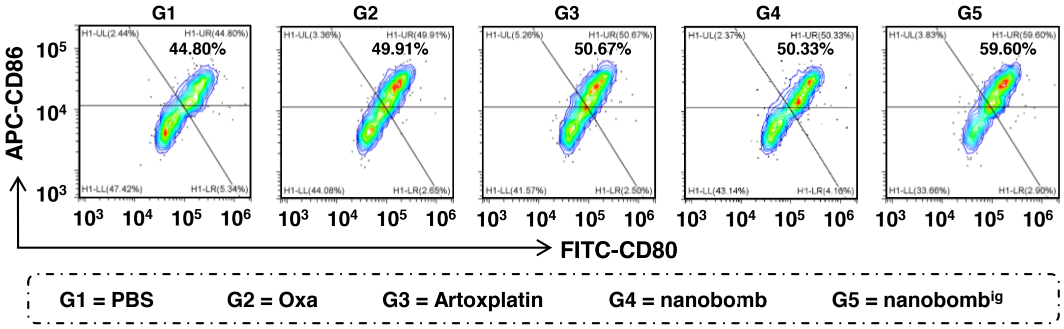

Figure S16. FCM analysis of BMDCs activation co-cultured with CT26 cells with various pretreatments.

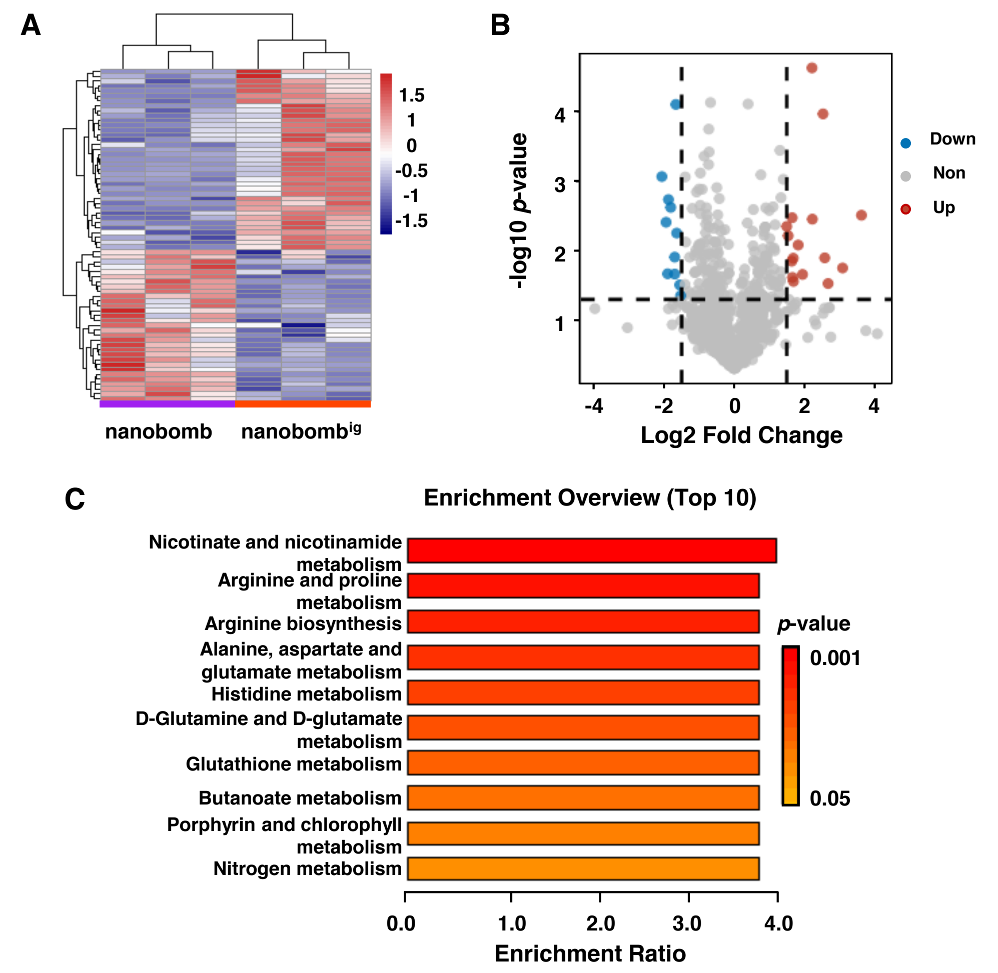

Figure S17. Metabolomic analysis of CT26 cells treated with nanobomb and nanobomb^{ig}. (A) Heatmap and (B) Volcano plot based on the different metabolites between the two groups. (C) Top ten KEGG pathway annotation of different metabolites between the two groups.

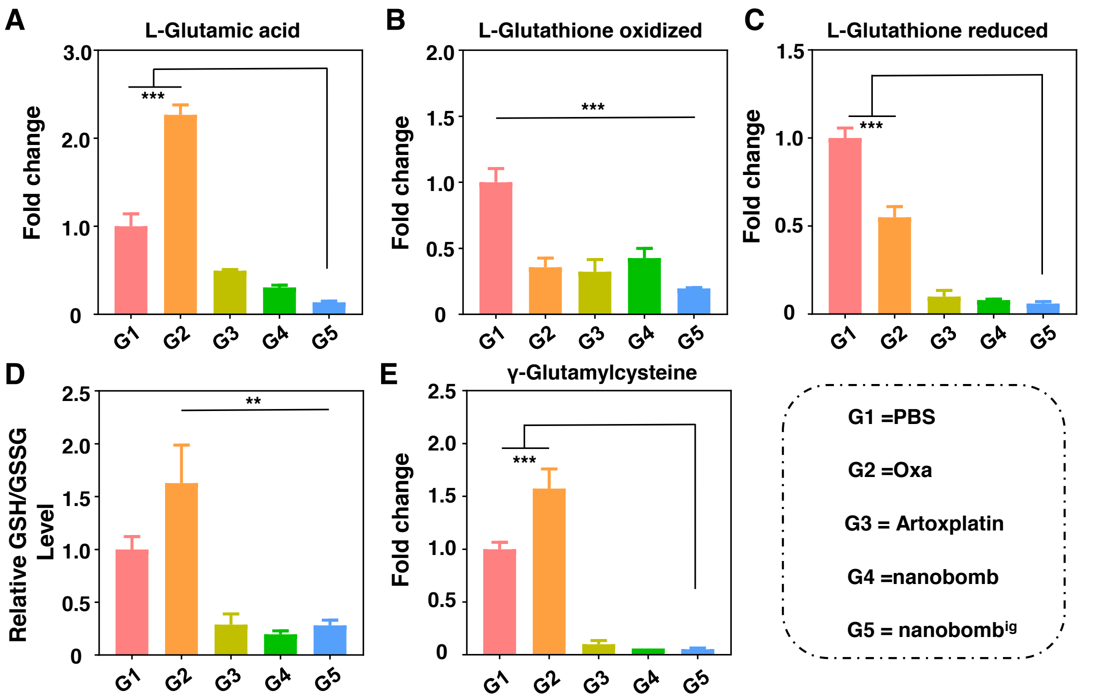

Figure S18. Different abundance of metabolites in Glutamine metabolic pathway. ***p* < 0.01, ****p* < 0.001.

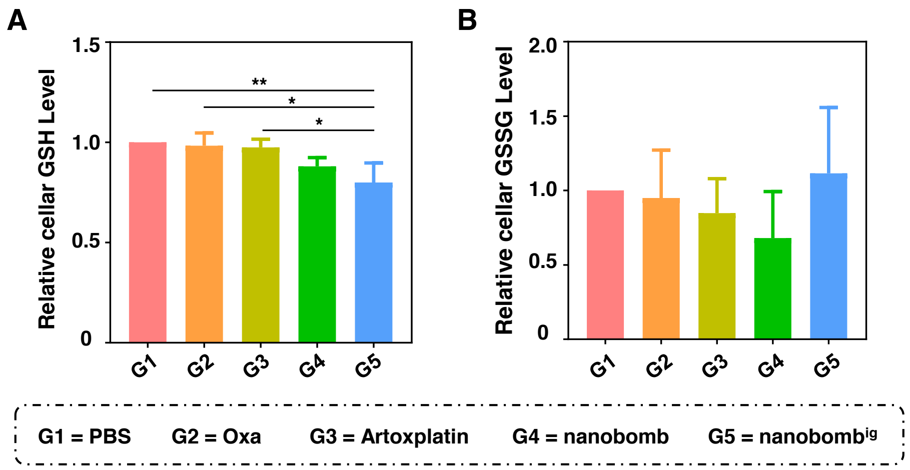

Figure S19. Relative GSH (A) and GSSG (B) levels in CT26 cells after various treatments. $*p < 0.05$, $**p < 0.01$.

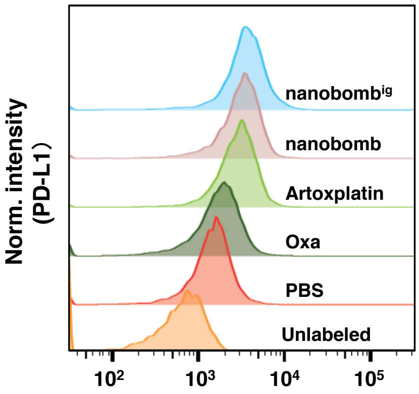

Figure S20. Representative flow cytometric profiles of membrane PD-L1 expression in CT26 cells.

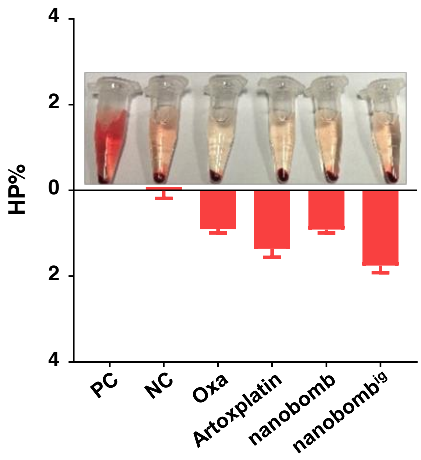

Figure S21. Photograph and the percentage of the hemolysis study in the mice blood sample upon various treatments. (n = 3). PC = Positive control, NC = Negative control, HP = Hemolytic percentage.

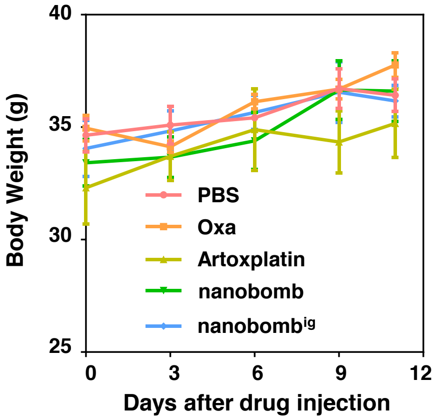

Figure S22. Body weight changes of KM mice treated with including PBS, Oxa, Artoxplatin， nanobomb and nanobomb^{ig} at 3 mg Pt kg^{-1} body weight (n = 3).

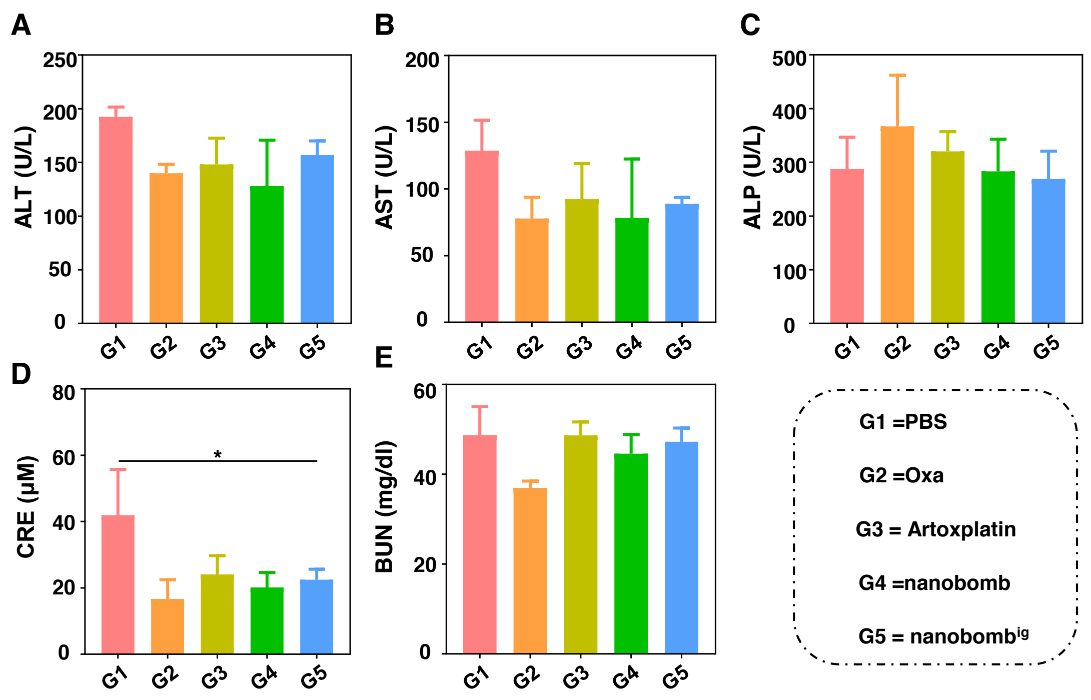

Figure S23. The *in vivo* biological safety evaluation of KM mice with various treatments for 11 days. (A-E) Serum biomarkers assess liver and kidney function, including alanine aminotransferase (ALT), aspartate aminotransferase (AST), alkaline phosphatase (ALP), creatinine (CRE) and urea nitrogen (BUN) (n = 3). $*p < 0.05$.

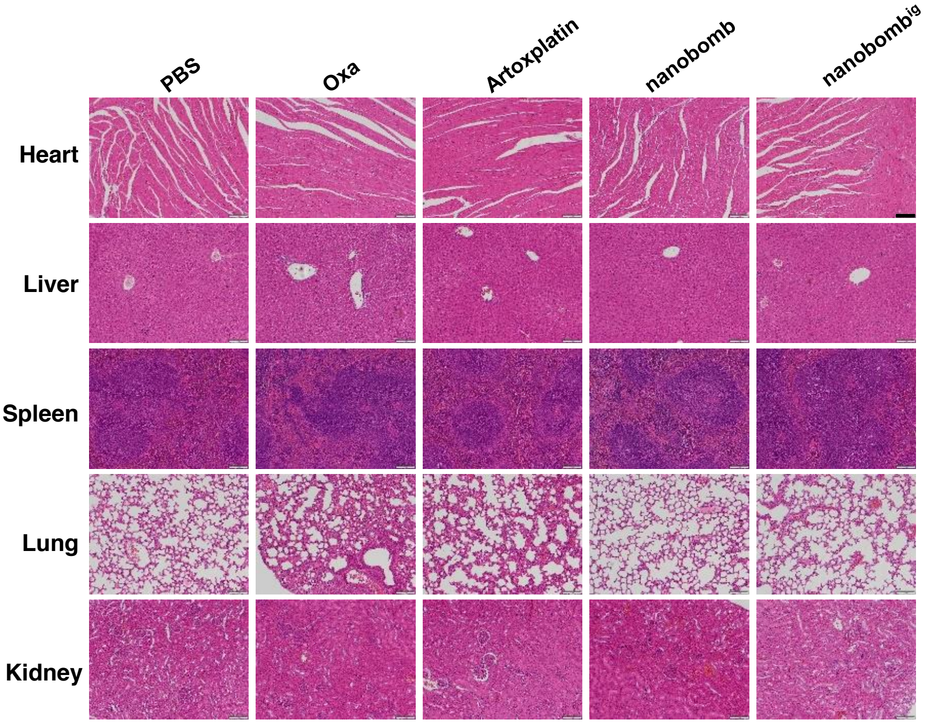

Figure S24. H&E staining of major organs (Heart, Liver, Spleen, Lung, and Kidney) of KM mice treated with including PBS, Oxa, Artoxplatin, nanobomb and nanobomb^{ig} at 3 mg Pt kg^{-1} body weight. Scale bar = 100 μm.

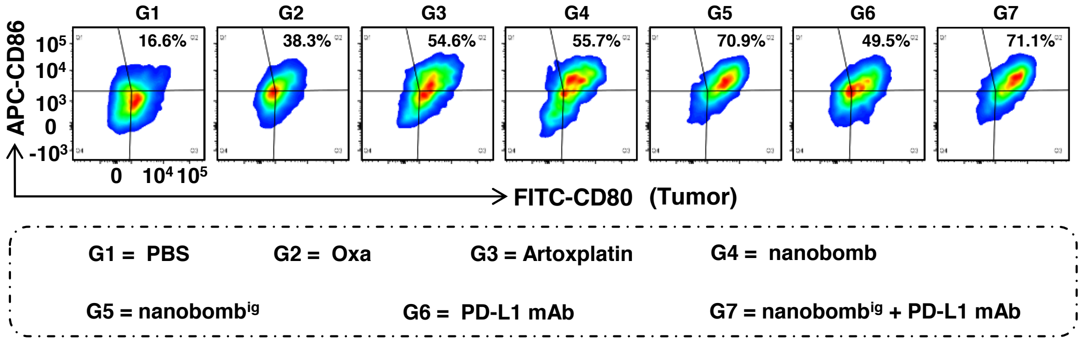

Figure S25. The representative FCM analysis images of DCs in tumor tissues (n = 3).

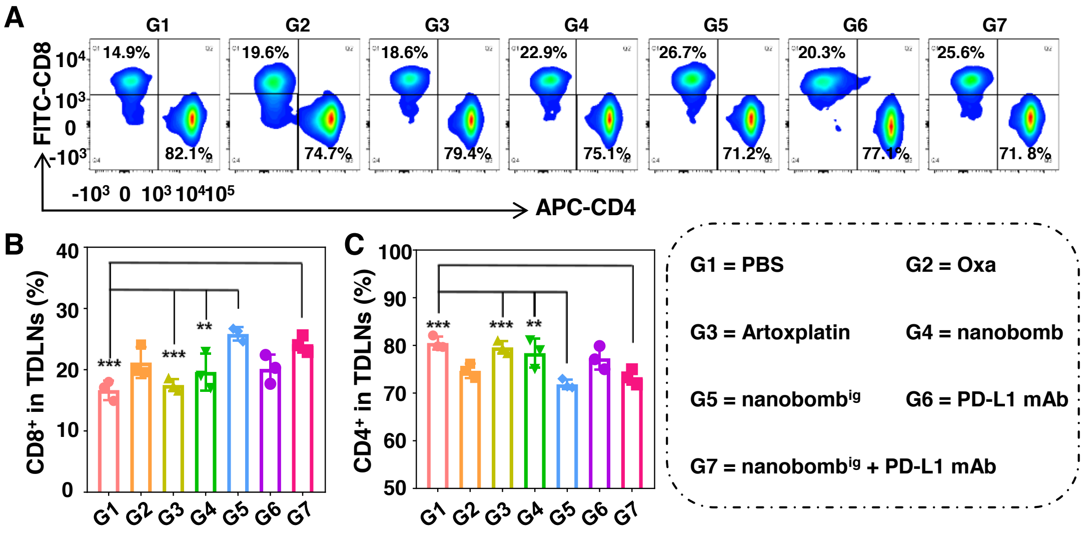

Figure S26. (A) The representative FCM analysis images of CD8$^+$ and CD4$^+$ T cells in TDLNs. The corresponding percentages of (B) CD8$^+$ and (C) CD4$^+$ T cells in TDLNs (n = 3). $^{**}p < 0.01$, $^{***}p < 0.001$.

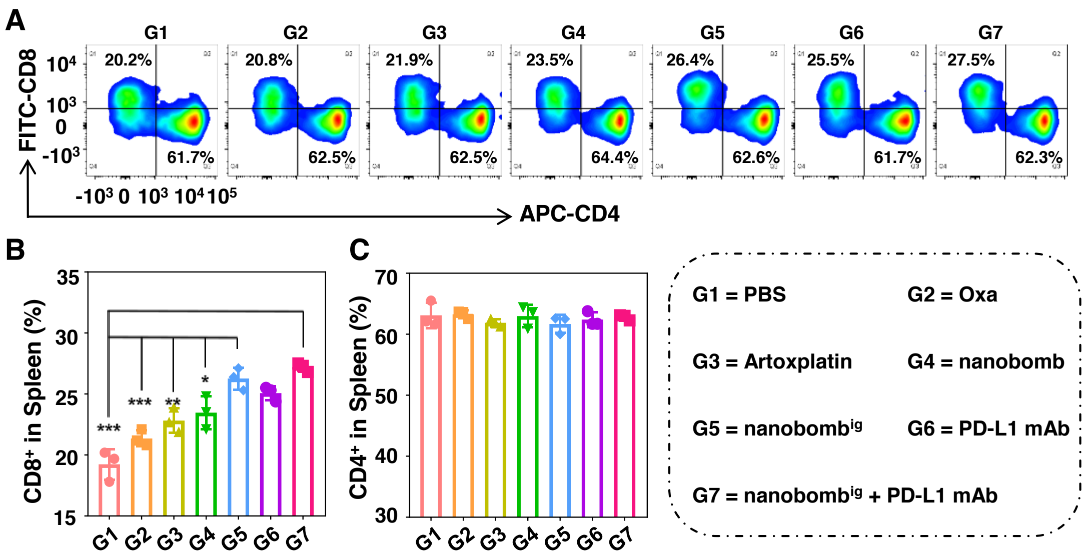

Figure S27. (A) The representative FCM analysis images of $CD8^+$ and $CD4^+$ T cells in spleen tissues. The corresponding percentages of (B) $CD8^+$ and (C) $CD4^+$ T cells in spleen tissues (n = 3). *$p < 0.05$, **$p < 0.01$, ***$p < 0.001$.

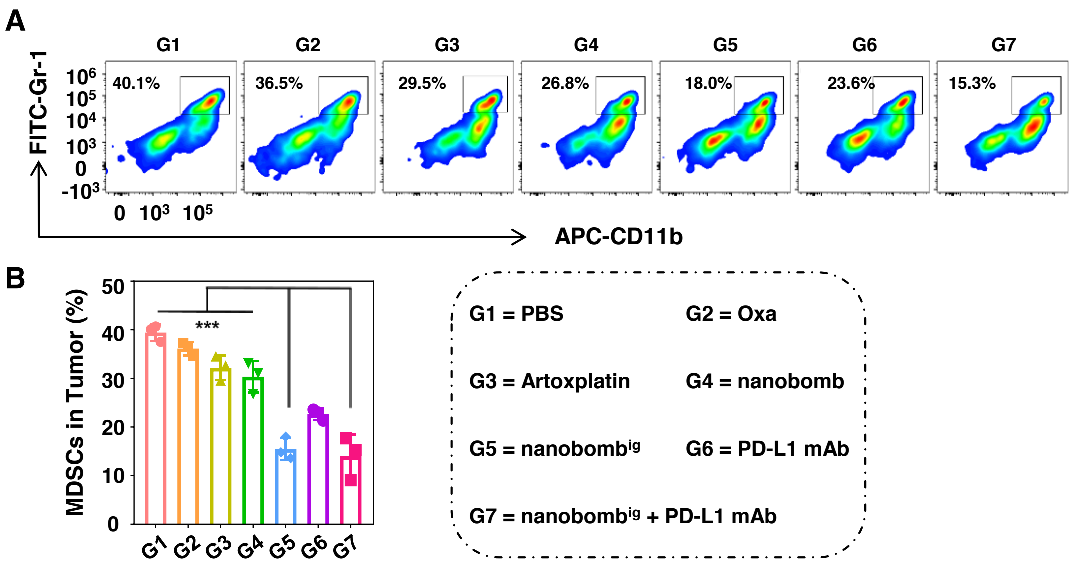

Figure S28. (A) The representative FCM analysis images of MDSCs ($CD45^+CD11b^+$ $Gr\text{-}1^+$) in tumor tissues. (B) The corresponding percentages of MDSCs in tumor tissues (n = 3). ***$p < 0.001$.

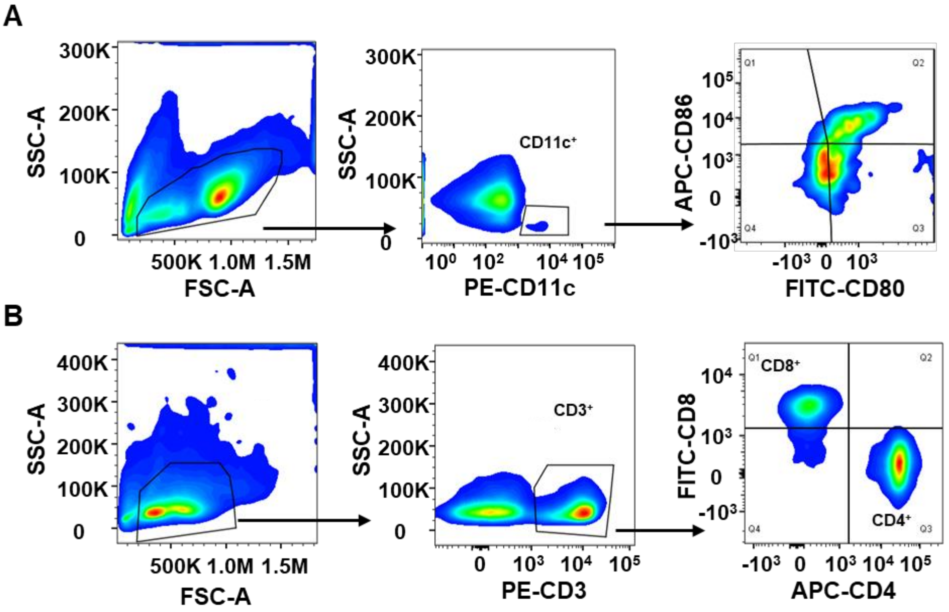

Figure S29. Gating strategies for FCM analysis of (A) DCs and (B) T cells in TDLNs.

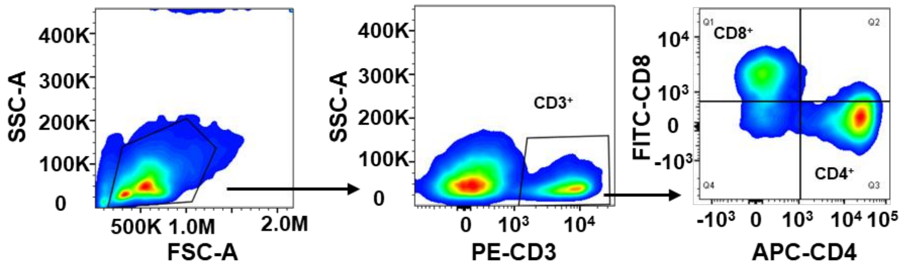

Figure S30. Gating strategies for FCM analysis of T cells in spleen tissues.

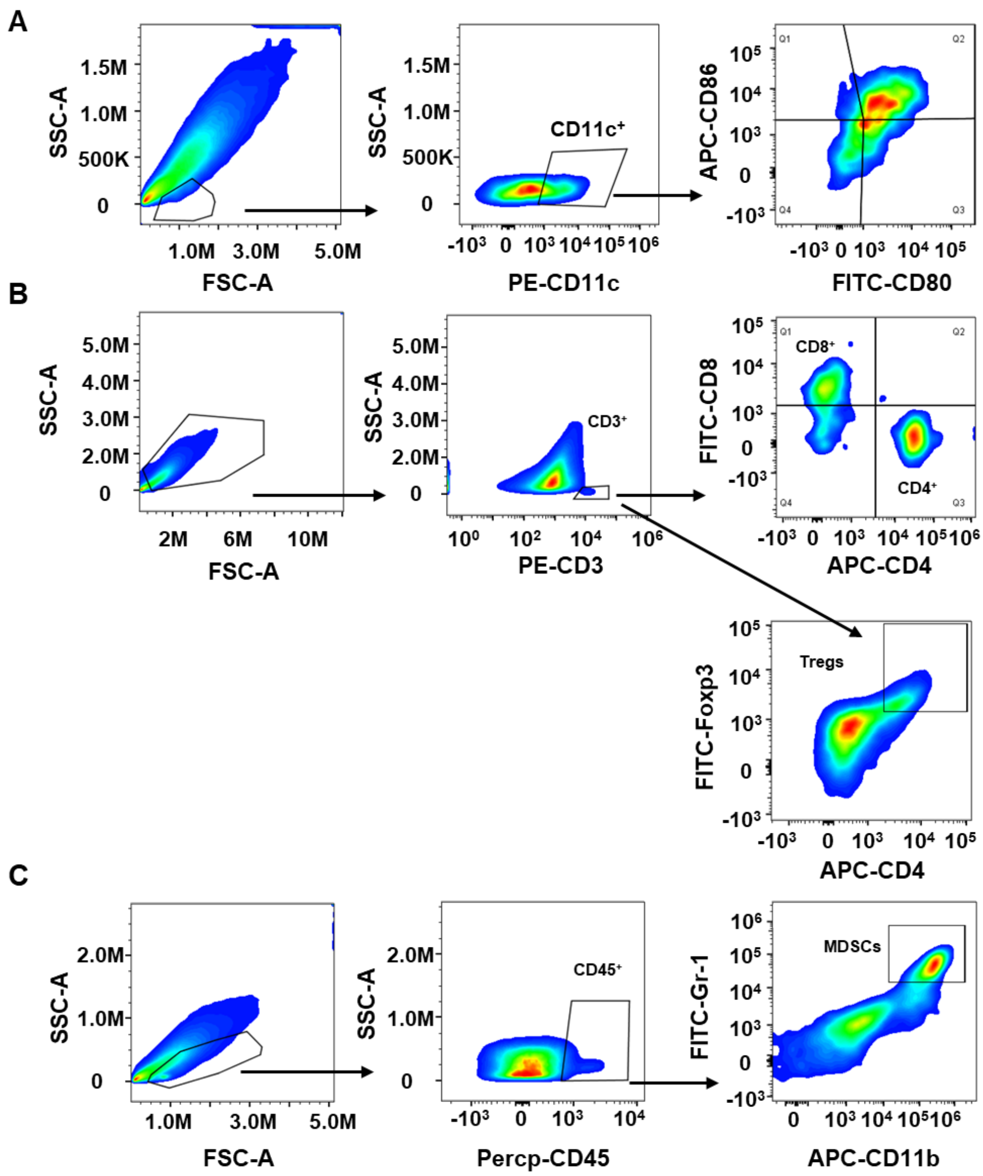

Figure S31. Gating strategies for FCM analysis of (A) DCs, (B) T cells, and (C) MDSCs in tumor tissues.

Table S1. Characterization of P1 by gel permeation chromatography (GPC) spectrum.

Polymer	Mn	Mw	MP	Mz	Mz+1	Mz/Mw
P1	14590	28363	23951	160986	624555	5.676

Mn: Number-average molecular weight; Mw: Weight-average molecular weight;

MP: Peak molecular weight; Mz: Size-average molecular weight.

References

[1] H. Xiao, R. Qi, T. Li, S. G. Awuah, Y. Zheng, W. Wei, X. Kang, H. Song, Y. Wang, Y. Yu, M. A. Bird, X. Jing, M. B. Yaffe, M. J. Birrer, P. P. Ghoroghchian, *J Am Chem Soc* **2017**, 139 (8), 3033.
[2] S. J. He, Z. M. Lin, Y. W. Wu, B. X. Bai, X. Q. Yang, P. L. He, F. H. Zhu, W. Tang, J. P. Zuo, *Acta Pharmacol Sin* **2014**, 35 (2), 219.
